# Atrial fibrillation and left atrial size and function: a Mendelian randomization study

**DOI:** 10.1038/s41598-021-87859-8

**Published:** 2021-04-19

**Authors:** Yordi J. van de Vegte, Joylene E. Siland, Michiel Rienstra, Pim van der Harst

**Affiliations:** 1 Department of Cardiology, University Medical Center Groningen, University of Groningen, Hanzeplein 1, P.O. Box 30.001, 9700 RB Groningen, The Netherlands; 2 Department of Genetics, University Medical Center Groningen, University of Groningen, Groningen, The Netherlands; 3 Department of Cardiology, Division of Heart and Lungs, University Medical Center Utrecht, University of Utrecht, Utrecht, The Netherlands

**Keywords:** Genetic association study, Atrial fibrillation, Cardiovascular genetics

## Abstract

Atrial fibrillation (AF) patients have enlarged left atria (LA), but prior studies suggested enlarged atria as both cause and consequence of AF. The aim of this study is to investigate the causal association between AF and LA size and function. In the UK Biobank, all individuals with contoured cardiovascular magnetic resonance data were selected. LA maximal volume (LA max), LA minimal volume (LA min), LA stroke volume and LA ejection fraction were measured and indexed to body surface area (BSA). Two-sample Mendelian randomization analyses were performed using 84 of the known genetic variants associated with AF to assess the association with all LA size and function in individuals without prevalent AF. A total of 4274 individuals (mean age 62.0 ± 7.5 years, 53.2% women) were included. Mendelian randomization analyses estimated a causal effect between genetically determined AF and BSA-indexed LA max, LA min, and LA ejection fraction, but not between AF and LA stroke volume. Leave-one-out analyses showed that the causal associations were attenuated after exclusion of rs67249485, located near *PITX2* gene. Our results suggest that AF causally increases LA size and decreases LA ejection fraction. The AF risk allele of rs67249485, located near the PITX2 gene, contributes strongly to these associations.

## Introduction

Atrial fibrillation (AF) is the most common cardiac arrhythmia worldwide, and many patients with AF develop an enlarged left atrium (LA)^1,2^. LA enlargement is associated with poorer prognosis of AF ablation outcomes and AF recurrences^3^, but may also increase the risk of stroke, adverse cardiovascular outcomes and death^4,5^.

LA enlargement is hypothesized to be a result of atrial remodeling, a persistent change in atrial structure or function^6,7^. However, pressure and/or volume overload commonly seen in conditions as hypertension, structural heart disease, mitral valve disease and heart failure may also induce change in atrial structure or function. As a consequence of atrial remodeling, it might trigger AF episodes, and then a vicious circle starts where AF episodes might trigger further atrial remodeling^8,9^. The degree of atrial remodeling can be assessed through measurement of LA volume with cardiovascular magnetic resonance (CMR) imaging^10^.

Co-existence of risk factors of AF and LA size and function makes it difficult to determine causality. Uncertainty exists if atrial remodeling is the cause or the consequence of AF. The hypothesis of a causal link between AF and LA volume may be tested by applying a Mendelian randomization approach (MR). Since AF associated genetic variants are randomly assigned at birth, a “naturally” randomized controlled trial can be performed, assuming that (1) genetic variants are reliably associated with AF, (2) genetic variants are independent of confounding factors and (3) genetic variants are only associated with LA volume through AF^11^. In present study, we use a two-sample MR approach to study the potential causal association between AF and LA size and function in the UK Biobank.

## Results

In the current study, 4274 individuals from the general population were included (mean age 62.0 ± 7.5, 53.2% women). The mean body mass index (BMI) was 26.6 (SD 4.4) kg/m^2^, the prevalence of hypertension and diabetes mellitus type II were 32.1% and 3.5%. Body surface area (BSA) indexed maximum LA volume (LA max), minimum LA volume (LA min) and LA stroke volume were 35.9 ± 10 ml/m^2^, 14.1 ml/m^2^ [Interquartile range (IQR) 10.9–17.9], and 21.1 ± 5.6 ml/m^2^, respectively. LA ejection fraction (LA EF) was on average 59.4 ± 8.3%. A total of 36 individuals (0.8%) developed AF during the median follow-up of 2.0 years (IQR 1.8–2.4). Additional information on the cohort is provided in Table 1. A total of 24 genetic variants were removed from MR analyses to reduce risk of weak instrument bias (F-statistic < 10) and 2 genetic variants were excluded during data harmonization. A total of 84 genetic variants were taken forward for further analyses. The total amount of genetic variants varies per outcome due to MR-Steiger filtering. Data supporting the genetic variants selection (F-statistics, data harmonization, Steiger filtering) and single genetic variant-estimates for all outcomes can be found in Supplementary Table 1.

**Table 1:** Baseline characteristics.

	Sample
No	4274
Age, y	62.0 ± 7.5
Sex, female, %	53.2
BMI, kg/m^2^	26.6 ± 4.4
Diabetes mellitus type 2, %	3.5
Hypertension, %	32.1
Prevalent atrial fibrillation, %	0
Incident atrial fibrillation, %	0.8
LA max (ml)	67.0 ± 20.0
LA max, indexed (ml/m^2^)	35.9 ± 10.0
LA min (ml)	26.0 (19.9–33.6)
LA min, indexed (ml/m^2^)	14.1 (10.9–17.9)
LA SV (ml)	39.2 ± 11.1
LA SV, indexed (ml/m^2^)	21.1 ± 5.6
LA EF (%)	59.4 ± 8.3
BSA (m^2^)	1.9 ± 0.2

Continuous variables are presented as mean ± SD, skewed variables (defined as -1 < skewness >1) as median (IQR min – IQR max) and binary variables as percentages. LA volumes were measured using the biplane method. BMI, denotes body mass index; LA, left atrium; SV, stroke volume; EF, ejection fraction; BSA, body surface area.

**Figure 1:**
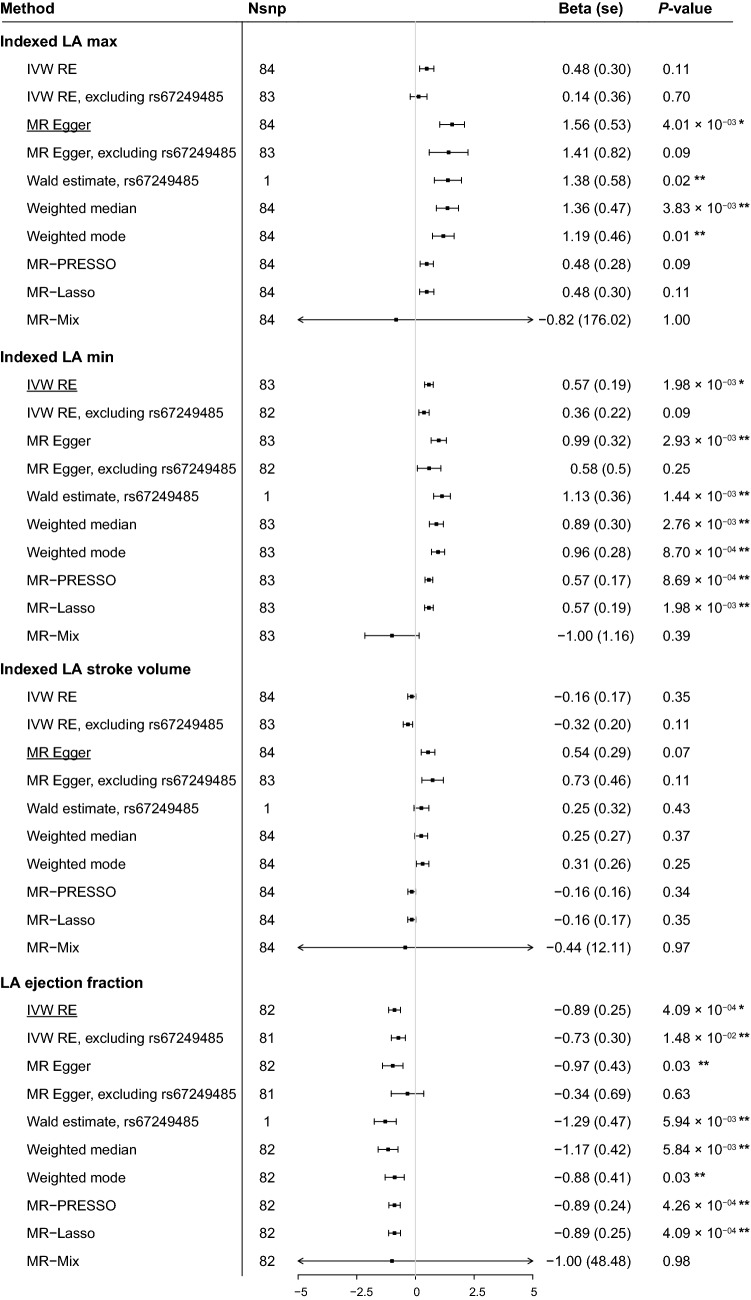
Summary MR estimates of the causal association between AF and LA size and function. The figure displays the MR estimates on the association between AF and body surface area indexed left atrial maximal volume (LA max), minimal volume (LA min), stroke volume and ejection fraction. Inverse-variance-weighted (random effects) model, MR-Egger, MR pleiotropy residual sum and outlier (MR-PRESSO), weighted median, weighted mode-based estimator and MR-Mix are shown. Outlier-corrected MR-PRESSO estimates are not included, since no genetic variants were removed in the MR-PRESSO analyses. On the X-axis, the beta coefficient and its upper and lower bound standard error are shown. The main analyses, i.e. inverse-variance-weighted random effects under the scenario of balanced horizontal pleiotropy or MR-Egger estimate under the scenario of unbalanced horizontal pleiotropy, are underlined per outcome. We considered a stringent two-sided Bonferonni corrected *P* < 0.05/7 outcomes statistically significant for the main analyses. Significant results for the main analysis are annotated with a single asterisk (*). A *P*-value threshold of *P* < 0.05 was adopted for the sensitivity MR analyses. Significant sensitivity MR analyses are annotated with a double asterisk (**). SE denotes standard error. The plot was made using the forestplot package (version 1.10.1, https://CRAN.R-project.org/package=forestplot) in R (version 3.6.3)^59^.

Results of the MR analyses between AF and indexed LA volumes and ejection fraction are shown in Fig. 1 and Supplementary Table 2. Additional information on the MR analyses of the unadjusted LA volumes can be found in Supplementary Table 2. Sensitivity analyses were performed to test whether the assumptions of the MR analyses were fulfilled (Supplementary Table 3). MR-Steiger directionality test indicated that the 84 genetic variants known to be associated with AF explained ~ 7% of AF variance. The genetic variants explained more of AF variance than indexed LA max volume (1.7%), indexed LA min volume (1.7%), indexed LA stroke volume (1.8%) and LA ejection fraction (2.0%) (Supplementary Table 3).

Using the Rücker framework, we found evidence for unbalanced horizontal pleiotropy in the MR estimates of indexed LA max and indexed LA stroke volume, indicated by significant Q–Q′ and MR-Egger intercepts (*P* < 0.05) (Supplementary Table 3). We therefore took forward the MR-Egger model as primary MR-method to assess the genetic association with indexed LA max and indexed LA stroke volume, whereas we adopted the inverse variance weighted random effects (IVW-RE) model for indexed LA min and LA ejection fraction. Using these models, we found evidence for a causal effect of genetic susceptibility to AF on indexed LA max (β = 1.56, SE = 0.53, *P* = 4.0 × 10^–3^), indexed LA min (β = 0.57, SE = 0.19, *P* = 2.0 × 10^–3^) and LA ejection fraction (β = − 0.89, SE = 0.25, *P* = 4.1 × 10^–4^) (Fig. 1). Weak-instrument bias was indicated within the MR-Egger estimate of AF on indexed LA max (I^2^_GX_ = 0.94). We did not find evidence for a causal association between genetic susceptibility to AF and indexed LA stroke volume (β = 0.54, SE = 0.29, *P* = 6.98 × 10^–2^). Scatter- and forest plots of the MR analyses between AF and all LA dimensions are provided in Supplementary Figs. 2–8.

Several sensitivity analyses were performed to test whether valid conclusions on causal inference could be made under different assumptions of possible underlying pleiotropy or instrumental invalidity. We investigated whether the results were consistent under the scenario where a relativity large portion of the genetic instruments is invalid using the weighted median approach. Using this approach, we found additional evidence for a significant causal estimate between genetic susceptibility to AF and indexed LA max (β = 1.36, SE = 0.47, *P* = 3.83 × 10^–3^), indexed LA min (β = 0.89, SE = 0.30, *P* = 2.8 × 10^–3^) and LA EF (β = − 1.17, SE = 0.42, *P* = 5.84 × 10^–3^). We then investigated whether the results were consistent under the scenario in which a small proportion of the genetic variants are outliers using the MR-Lasso approach. Using this approach, we find the genetic associations between AF and indexed LA min (β = 0.57, SE = 0.19, *P* = 1.98 × 10^–3^) as well as LA ejection fraction (β = − 0.89, SE = 0.25, *P* = 4.09 × 10^–4^) to be robust to this scenario. However, the association between genetic susceptibility to AF and indexed LA max (β = 0.48, SE = 0.30, *P* = 1.13 × 10^–1^) was attenuated (Fig. 1).

We examined which genetic variant(s) drove the attenuation of the association between genetic susceptibility to AF and LA size and function by performing leave-one-out analyses. Results of the leave-one-out analyses using an IVW and MR-Egger approach are provided in Supplementary Table 4 and can be visually inspected in Supplementary Figs. 9–15. We observed that the MR-Egger estimate of AF on indexed LA max was attenuated after exclusion of rs67249485 (β = 1.41, SE = 0.82, *P* = 9.05 × 10^–2^), a genetic variant located on the long arm of chromosome 4 in the proximity of the *PITX2* gene. However, the Wald estimate of rs67249485 did show a significant association for indexed LA max (β = 1.38, SE = 0.58, *P* = 1.65 × 10^–2^). The results are shown in Fig. 1. The leave-one-out analyses also showed an attenuation of IWR-RE estimates after exclusion of rs67249485 for indexed LA min (β = 0.36, SE = 0.22, *P* = 1.00 × 10^–1^), and LA EF (β = − 0.73, SE = 0.30, *P* = 1.52 × 10^–2^). The Wald statistics for the association between rs67249485 and indexed LA min (β = 1.13, SE = 0.36, *P* = 1.44 × 10^–3^) as well as LA ejection fraction (β = − 1.29, SE = 0.47, *P* = 5.94 × 10^–3^) were significant (Fig. 1).

We performed several quality controls to gain insights in the statistical validity of rs67249485 driving the association between genetic susceptibility to AF and LA dimensions and functions. Histograms of LA dimension distributions per AF increasing T allele showed absence of outliers which could drive current MR estimates (Supplementary Fig. 16). The genetic variant rs67249485 explained more variance for AF (MR-Steiger R^2^ = 1.58%) than for any LA size or function, which ranged up to a maximum explained variance of 0.23% for LA min. This indicates that the Wald estimates assessed the true causal direction (Supplementary Table 1).

Lastly, we performed multivariable MR analyses to assess whether the described genetic associations between AF and LA size and function are independent of blood pressure as it can affect both AF^12^ and LA size and function^13,14^. In brief, all multivariable Mendelian randomization analyses were similar to the univariable results. For example, the main MR-Egger analyses of AF on index LA max (β = 1.56, SE = 0.53, *P* = 4.0 × 10^–3^) had similar effect estimates as in the multivariable MR in which we corrected for systolic blood pressure (β = 1.68, SE = 0.53, *P* = 1.6 × 10^–3^). Please see Supplementary Table 2 for the full results and Supplementary Table 3 for the sensitivity analyses.

The MR analyses for the non-indexed LA volumes are provided in Supplementary Tables 1–4. The results were consistent to the results on the indexed LA volumes. The MR analyses for LA min (indexed and non-indexed) were repeated using genetic variant-outcome effect estimates obtained from their log-transformed equivalents to account for right skewness. Results were comparable to the primary analyses (Supplementary Table 2).

## Discussion

Our study provides evidence to support the hypothesis that genetically susceptibility to AF increases indexed LA max, LA min and decreases LA ejection fraction. We pinpoint that rs67249485, near the *PITX2* gene, is the driver of the association with indexed LA max and LA min and contributes strongly to the association with LA ejection fraction. However, we did not find evidence for a causal association between AF and LA stroke volume.

Our primary analyses indicate that genetic susceptibility to AF causally increases indexed LA max and LA min. A causal association between AF and LA stroke volume was not established. One potential explanation for this discrepancy is that AF increases indexed LA max and indexed LA min in a similar degree, nullifying the effect on LA stroke volume. Another potential explanation is that a larger passive conduit function of the LA could compensate for a decreased pump function at larger maximal LA volume through the Frank-Starling law^15,16^. This would result in similar LA stroke volume and lower LA ejection fraction^15,16^. In fact, we do find that genetic susceptibility to AF is associated with decreased LA ejection fraction.

The described associations between AF and indexed LA max, indexed LA min and LA EF were attenuated after exclusion of rs67249485, located in an intergenic region near the *PITX2* gene^17^. Our results suggest rs67249485 to be the main driver of the genetic association between AF and indexed LA max and LA min as the main analyses were nullified after exclusion of rs67249485, while the Wald estimates of rs67249485 was significant. We still find a causal estimate between genetic susceptibility to AF and LA EF after exclusion of this variant, which may suggest that other genetic variants may also contribute to the genetic association between AF and LA EF. The validity of rs67249485 as important driver in the association between AF and LA size and function is statistically supported by several sensitivity analyses which indicate that the large effect of this genetic variants is very unlikely caused by measurement error, uneven population distribution or incorrect direction of causality. The biological role of *PITX2* in AF development has been extensively studied and many potential mechanisms have been suggested, including deviations in LA myocyte automaticity, impaired response to oxidative stress, inflammation and a role in the embryonic development of the heart^18–21^. The *PITX2* gene does not only increase the risk of AF development, but has been suggested as a determinant in the success of pulmonary vein ablation in preventing AF recurrence as well^22^*.* Our results provide evidence for another possible biological consequence of *PITX2,* as we show that LA volumes increase and LA ejection fraction decreases through the AF increasing T allele of rs67249485. However, further experimental validation is needed to investigate details of the mechanisms underlying the association of rs67249485, *PITX2*, AF and LA size and function.

One cardiovascular risk factor that could potentially affect our results is hypertension, as blood pressure is known to affect both AF and LA size and function^12–14^. We therefore performed additional multivariable MR analyses and find that the described associations between AF and LA size and function are independent of systolic blood pressure, diastolic blood pressure and pulse pressure^23^.

Our study has several strengths. The strengths include the use state-of-art genetic and CMR data. The MR design is less susceptible to confounding and strongly contributes to previous work in the field^24^. We excluded individuals with known prevalent AF and the MR was designed to study the effect of increased AF risk on LA dimensions before onset of the disease. Extensive sensitivity analyses were performed to further reduce the risk of pleiotropy and reversed causation and support our hypothesis.

Some limitations should be noted as well. First, the genetic variants used as proxy for AF explained approximately 7% of AF variance, which is a proportion of total genetic variance of 62% that has been suggested in a previous twin study^25^. We note that we did not include all previously established genetic variants associated with AF as the UK Biobank was used as discovery cohort in the most recent GWAS of AF^17^. We therefore took forward the largest set of genetic variants using effect sizes obtained without the UK Biobank to limit overlap of the exposure and outcome cohorts. In addition, a part of the heritability of AF and LA size is still unknown and there remains a gap between SNP-based and classic heritability estimates^26^. Several reasons for the missing heritability have been hypothesized, including the focus of GWAS on common genetic variants and the inclusion of individuals that are mainly from European descent^26^. In addition, GWAS assumes an additive model which overlook epistatic effects and possible interactions between genetics and the environment^26^. Further research to the genetics of AF by studying whole exome sequencing data^27,28^, expanding the reference genome with other ancestries^29^, gene–gene^30,31^ and gene–lifestyle interaction^32,33^ could increase our insights in AF and consequently the certainty of the described genetic association between AF and LA size and function. We did not have data on LA volume at the onset of atrial contraction and were therefore unable to differentiate the effect of AF on the LA conduit and pump function separately. Pleiotropy cannot be ruled out completely despite rigorous sensitivity analyses. We were unable to perform a bidirectional MR to further entangle the cause and consequence in the association between AF and LA size and function as the current cohort is too small to identify robustly associated genetic variants. Lastly, the AF associated variants were obtained from a multi-ethnic GWAS meta-analysis, while the outcome cohort included individuals that were mainly from European descent. Population stratification could introduce confounding in the MR analyses through hidden population structure if the ancestry is correlated with both the phenotypes and genotypes^34^. However, we believe this to be unlikely given the stringent adjustments for genetic ancestry in the GWAS of AF and in the regression analyses on atrial size and function^35^.

In conclusion, we provide evidence that a higher genetic susceptibility to AF increases indexed LA max and LA min, while it decreases LA EF. We pinpoint that the genetic variant rs67249485, near the *PITX2* gene, drives the association between AF and indexed LA max and LA min and contributes strongly to the genetic association between AF and LA EF. The association between AF and LA EF was robust to multiple sensitivity analyses and indicate that genetic susceptibility to AF causally decreases LA EF.

## Methods

### Study population

The UK Biobank is a large, population-based cohort that included 503,325 individuals via general practitioners of the UK National Health Service (NHS) between 2006 and 2010. Informed consent was obtained from all included individuals and the North West Multi-centre Research Ethics Committee approved of the study and the North West Multi-centre Research Ethics Committee approved of the study^36^. The UK Biobank study has been carried out in accordance with relevant guidelines and regulations and has approval from all relevant institutional review boards, including the North West Multi-centre Research Ethics Committee for the UK, the National Information Governance Board for Health and Social Care for England and Wales, and the Community Health Index Advisory Group for Scotland^36^. Hospital episode statistics were available up to 31-03-2017 for English participants, 29-02-2016 for Walsh participants and 31-10-2016 for Scottish participants. Individuals with contoured CMR data, as previously performed by Petersen et al*.*, were included in the current study^37^. Individuals were excluded in case of missing information on body surface area or any covariates (please see below), failure of genetic quality control (including heterozygosity, high missingness and a discrepancy between reported and inferred gender), familial relatedness, or a medical history of mitral valve disease, heart failure, valvular surgery, pulmonary hypertension or prevalent AF at the time of CMR. Definitions of prevalent incident and incident disease are presented in Supplementary Table 1 and a flowchart depicting the study sample selection is shown in Supplementary Fig. 1.

### Left atrial size and function

CMR protocol and image analyses of left atrial dimensions have been described previously^10^. In brief, all CMR examinations in UK Biobank were performed on a clinical wide bore 1.5 T scanner (MAGNETOM Aera, Sygno Platform VD13A, Siemens Healthcare, Erlangen, Germany) in Cheadle, United Kingdom. The LA dimensions were manually analyzed by two core laboratories based in London and Oxford and the returned volumes were used in the current study^37^. In each CMR examination, endocardial LA contours were manually traced at end-systole (maximal LA area) and end-diastole (minimal LA area) in the HLA (4-chamber) view and VLA (2-chamber) view. The biplane method was applied to calculate maximal and minimal areas. Maximal LA volume (LA max volume) is defined as the end of left ventricular systole. Minimal LA volume (LA min volume) is defined as the end of left ventricular diastole. LA stroke volume and LA ejection fraction were calculated as followed: LA stroke volume = (*LA max* − *LA min*) and LA ejection fraction = 100 × (*LA max* − *LLA min*)/(*LA max*).

LA volumes (LA max and LA min and LA stroke volume) were indexed to body surface area (BSA) to account for body size as well as gender differences^12^. We took forward these seven outcomes to evaluate the association between AF associated genetic variants and LA size and function. As sensitivity analyses, we log-transformed LA min (indexed and non-indexed) to account for right skewness.

### Genotype and imputed data

The Wellcome Trust Centre for Human Genetics performed genotyping and quality control before imputation in the individuals of UK Biobank, and imputed to HRC v1.1 panel. The quality control of samples and variants, and imputation was previously described in detail^38^.

### Genetic variants: atrial fibrillation

In this study, 111 genetic variants associated with AF (*P*-value < 5 × 10^–8^) from the prior GWAS of Nielsen et al*.* were selected as genetic instruments in current analyses^39^. The effect sizes of the genetic variants associated with AF within the independent cohorts of the Broad AF Study, BBJ, EGCUT, PHB, SiGN and the Vanderbilt AF Registry published by Roselli et al*.* were used (number of cases = 32,957, number of controls = 83,546)^17^. We opted for this approach to obtain one of the largest sets of robust AF genetic instruments, while also being able to use effect sizes that were independent of the UK Biobank to limit overlap of the exposure and outcome cohorts. One genetic variant (rs17005647) was a priori removed as we were unable to precisely calculate the beta with the provided odds ratio of 1.0.

### Genetic variants: left atrial size and function

Effect estimates of the AF associated genetic variants on LA size and function were obtained from all individuals included in the current study. Effect sizes were obtained by performing linear regression analyses on LA size and function, which were corrected for age during the imaging visit, sex, 30 principal components and genotyping array.

### Genetic variants: blood pressure traits

Effect estimates of the AF associated genetic variants on systolic blood pressure, diastolic blood pressure and pulse pressure were obtained from a cohort of 408,212 unrelated individuals from the UK Biobank that were not included in the estimates of LA size and function. Systolic and diastolic blood pressure values were obtained during the baseline visit through two automated and/or two manual blood pressure measurements and the average of all measurements was used. The automated measurements were corrected according to previously described methodology^40^. Pulse pressure was calculated by subtracting diastolic from systolic blood pressure. Blood pressure altering medication use was taken into account by adding respectively 15, 10 mmHg and 5 mmHg to the blood pressure trait^41^. Effect sizes were obtained by performing linear regression analyses, which were corrected for age during the baseline visit, sex, 30 principal components and genotyping array.

### Mendelian randomization analysis

The genetic variants were tested for weak instrument bias (F-statistic) and reversed causation (MR-Steiger). F-statistics were calculated per genetic variant using the following formula: *F* = *R*^2^(*n* − 2)/(1 − *R*^2^)*.* Here, *n* is the sample size of the exposure and R^2^ is the amount of variance of the exposure explained by the genetic variant^42^. An F-statistic < 10 was considered to indicate weak-instrument bias and these genetic variants were removed from further analyses. Reversed causation was assessed through MR-Steiger filtering and genetic variants with a significantly higher (*P* < 0.05) R^2^ for the outcome than for the exposure were removed^43^. The R^2^ for AF (on the liability scale)^44^ and linear outcomes^45^ were calculated based on the summary statics provided in Supplementary Table 1 using previously established formulae.

MR estimates were generated using inverse-variance weighted random effects meta-analysis. The Rucker framework was applied to assess heterogeneity and thus potential pleiotropy within the MR effect estimates^46^. Balanced horizontal pleiotropy was assessed by calculating Cochran’s Q (*P* < 0.05) and *I*^2^ index (> 25%) as indicators of heterogeneity within the IVW model^47^. Potential unbalanced pleiotropy was assessed by performing MR-Egger regression as the MR-Egger allows for a non-zero intercept^48^. The Rucker framework than assesses the difference between heterogeneity within the IVW effect estimate (Cochran’s Q) and heterogeneity within the MR-Egger regression (Rucker’s Q), called Q–Q′. A significant Q–Q′ (*P* < 0.05), in combination with a significant non-zero intercept of the MR-Egger regression (*P* < 0.05), was considered to indicate unbalanced horizontal pleiotropy. Under this scenario, we report the MR-Egger effect estimates as it provides a causal estimate if the general InSIDE (Instrument Strength Independent of Direct Effect) assumption holds^48^. Weak instrument bias within the MR-Egger regression was assessed by I^2^_GX_. An I^2^_GX_ of > 95% was considered low risk of weak instrument bias within the MR-Egger estimates^49^. The main analysis consisted of either the IVW-RE (under the scenario of balanced horizontal pleiotropy) or the MR-Egger estimate (under the scenario of unbalanced horizontal pleiotropy).

Additional sensitivity analyses included the Mendelian randomization-Pleiotropy Residual Sum and Outlier (MR-PRESSO)^50^, MR-Lasso^51^, leave-one-out analyses^52,53^, weighted median^54^, weighted mode^55^ and MR-Mix^56^, multivariable MR-IVW^23^, multivariable MR-Egger^57^ and multivariable MR-PRESSO^50^. These all have their own strength and weaknesses and jointly provide information on the possibility of a true causal relationship. Outlier robust methods include MR-PRESSO (excludes outliers), leave-one-out analyses (excludes genetic variants one by one and reperforms IVW and MR-Egger analyses) and MR-Lasso (downweights outliers). Weighted median (majority valid), weighted mode and MR-MIX (plurality valid) generally have the potential to estimate true causal effects when larger proportions of genetic variants violate MR assumptions (generally at the cost of power). The multivariable MR-IVW^23^, multivariable MR-Egger^57^ and multivariable MR-PRESSO^50^ analyses were performed to correct for the potential influence of systolic blood pressure, diastolic blood pressure and pulse pressure in the causal association between AF and LA size and function^12–14^. Effect estimates for blood pressure traits were obtained in an independent cohort from the UK Biobank (See: Genetic variants: blood pressure traits). Weak instrument bias within the multivariable MR setting was considered unlikely if Q_x1_ and Q_x2_ were larger than the critical value at the χ^2^, calculated by subtracting one degree of freedom from the amount of SNPs at a *P* value of 0.05^23^. Potential pleiotropy within the multivariable MR setting was assessed using the Q_a_, which was considered to indicate potential pleiotropy when larger than the critical value on the χ^2^ distribution as calculated by the amount of SNPs minus two degrees of freedom at a *P* value of 0.05^23^. A multivariable MR-Egger intercept with a *P* value < 0.05 was considered prove of unbalanced horizontal pleiotropy and the MR-Egger regression to provide a robust causal estimate^57^.

Causal effect estimates are reported in β values, since LA volumes and fractions are continuous variables. The main analyses were considered significant at a Bonferonni corrected α = 0.05/7 outcomes. For the sensitivity analyses, we adapted α = 0.05 to ascertain statistical significance when replicating the findings of the main analysis. Continuous variables are displayed as mean ± standard deviation when normally distributed and as median and interquartile ranges when skewed. Categorical variables are displayed as percentages. Regression analyses to obtain genetic variant-outcome associations were performed using statistical software STATA 15 (StataCorp LP)^58^. MR analyses were performed using R (version 3.6.3)^59^, the TwoSampleMR package 0.5.3^60^, MR-PRESSO (version 1.0)^50^, MR-Lasso^51^, MR-mix (version 0.1.0)^56^, MendelianRandomization (version 0.5.0)^61^ and MVMR (version 0.3)^23^.

## Supplementary Information

Supplementary Information 1.

Supplementary Information 2.

## Data Availability

The data that support the findings of this study are available from the corresponding author upon reasonable request.

## References

[1] Chugh SS (2014). Worldwide epidemiology of atrial fibrillation: A global burden of disease 2010 study. Circulation.

[2] Kirchhof, P. *et al.* 2016 ESC Guidelines for the management of atrial fibrillation developed in collaboration with EACTS. *Eur. Heart J. ***37**, 2893–2962 (2016). 10.1093/eurheartj/ehw210 27567408

[3] Zhuang J (2012). Association between left atrial size and atrial fibrillation recurrence after single circumferential pulmonary vein isolation: A systematic review and meta-analysis of observational studies. Eurospace..

[4] Benjamin EJ, D’Agostino RB, Belanger AJ, Wolf PA, Levy D (1995). Left atrial size and the risk of stroke and death: The Framingham Heart Study. Circulation.

[5] Flaker GC (1995). Clinical and echocardiographic features of intermittent atrial fibrillation that predict recurrent atrial fibrillation. Am. J. Cardiol..

[6] Nattel S, Burstein B, Dobrev D (2008). Atrial remodeling and atrial fibrillation: Mechanisms and implications. Circ. Arrhythm. Electrophysiol..

[7] Casaclang-Verzosa G, Gersh BJ, Tsang TSM (2008). Structural and functional remodeling of the left atrium. Clinical and therapeutic implications for atrial fibrillation. J. Am. Coll. Cardiol..

[8] Allessie, M. A. Atrial electrophysiologic remodeling: Another vicious circle? In *Journal of Cardiovascular Electrophysiology* vol. 9 1378–1393 (Futura Publishing Company Inc., 1998). 10.1111/j.1540-8167.1998.tb00114.x 9869538

[9] Wijffels MCEF, Kirchhof CJHJ, Dorland R, Allessie MA (1995). Atrial fibrillation begets atrial fibrillation: A study in awake chronically instrumented goats. Circulation.

[10] Lang RM (2015). Recommendations for cardiac chamber quantification by echocardiography in adults: An update from the American Society of Echocardiography and the European Association of Cardiovascular Imaging. J. Am. Soc. Echocardiogr..

[11] Davey Smith G, Hemani G (2014). Mendelian randomization: Genetic anchors for causal inference in epidemiological studies. Hum. Mol. Genet..

[12] Dzeshka MS, Shantsila A, Shantsila E, Lip GYH (2017). Atrial fibrillation and hypertension. Hypertension.

[13] Eshoo S, Ross DL, Thomas L (2009). Impact of mild hypertension on left atrial size and function. Circ. Cardiovasc. Imaging.

[14] Vaziri SM, Larson MG, Lauer MS, Benjamin EJ, Levy D (1995). Influence of blood pressure on left atrial size: The Framingham Heart Study. Hypertension.

[15] Anwar AM, Geleijnse ML, Soliman OII, Nemes A, Ten Cate FJ (2007). Left atrial Frank–Starling law assessed by real-time, three-dimensional echocardiographic left atrial volume changes. Heart.

[16] Tenekecioglu E (2014). Disturbed left atrial function is associated with paroxysmal atrial fibrillation in hypertension. Arq. Bras. Cardiol..

[17] Roselli C (2018). Multi-ethnic genome-wide association study for atrial fibrillation. Nat. Genet..

[18] Mommersteeg MTM (2007). Pitx2c and Nkx2-5 are required for the formation and identity of the pulmonary myocardium. Circ. Res..

[19] Kirchhof P (2011). PITX2c is expressed in the adult left atrium, and reducing Pitx2c expression promotes atrial fibrillation inducibility and complex changes in gene expression. Circ. Cardiovasc. Genet..

[20] Wang J (2010). Pitx2 prevents susceptibility to atrial arrhythmias by inhibiting left-sided pacemaker specification. Proc. Natl. Acad. Sci. U. S. A..

[21] Tao G (2016). Pitx2 promotes heart repair by activating the antioxidant response after cardiac injury. Nature.

[22] Shoemaker MB (2020). Genetic susceptibility for atrial fibrillation in patients undergoing atrial fibrillation ablation. Circ. Arrhythm. Electrophysiol..

[23] Sanderson E, Davey Smith G, Windmeijer F, Bowden J (2019). An examination of multivariable Mendelian randomization in the single-sample and two-sample summary data settings. Int. J. Epidemiol..

[24] Smith GD, Ebrahim S (2003). ‘Mendelian randomization’: Can genetic epidemiology contribute to understanding environmental determinants of disease?. Int. J. Epidemiol..

[25] Christophersen IE (2009). Familial aggregation of atrial fibrillation: A study in danish twins. Circ. Arrhythm. Electrophysiol..

[26] Manolio TA (2009). Finding the missing heritability of complex diseases. Nature.

[27] Petersen, B. S., Fredrich, B., Hoeppner, M. P., Ellinghaus, D. & Franke, A. Opportunities and challenges of whole-genome and -exome sequencing. *BMC Genet. ***18**, Article 14 (2017). 10.1186/s12863-017-0479-5 PMC5307692 28193154

[28] Wainschtein P (2019). Recovery of trait heritability from whole genome sequence data. bioRxiv.

[29] Sherman RM (2019). Assembly of a pan-genome from deep sequencing of 910 humans of African descent. Nat. Genet..

[30] Hu JK, Wang X, Wang P (2014). Testing gene–gene interactions in genome wide association studies. Genet. Epidemiol..

[31] Cordell HJ (2009). Detecting gene–gene interactions that underlie human diseases. Nat. Rev. Genet..

[32] Abdullah Said M, Verweij N, Van Der Harst P (2018). Associations of combined genetic and lifestyle risks with incident cardiovascular disease and diabetes in the UK biobank study. JAMA Cardiol..

[33] Said, M. A. *et al.* Contributions of interactions between lifestyle and genetics on coronary artery disease risk. *Curr. Cardiol. Rep.* **21**, Article 89 (2019). 10.1007/s11886-019-1177-x PMC6661028 31352625

[34] Vanderweele TJ, Tchetgen Tchetgen EJ, Cornelis M, Kraft P (2014). Methodological challenges in Mendelian randomization. Epidemiology.

[35] Price AL (2006). Principal components analysis corrects for stratification in genome-wide association studies. Nat. Genet..

[36] *UK Biobank Ethics and Governance** Framework*. https://www.ukbiobank.ac.uk/wp-content/uploads/2011/05/EGF20082.pdf (2012). Accessed 7 Mar 2019.

[37] Petersen SE (2017). Reference ranges for cardiac structure and function using cardiovascular magnetic resonance (CMR) in Caucasians from the UK Biobank population cohort. J. Cardiovasc. Magn. Reson..

[38] Bycroft C (2018). The UK Biobank resource with deep phenotyping and genomic data. Nature.

[39] Nielsen JB (2018). Genome-wide study of atrial fibrillation identifies seven risk loci and highlights biological pathways and regulatory elements involved in cardiac development. Am. J. Hum. Genet..

[40] Stang A (2006). Algorithms for converting random-zero to automated oscillometric blood pressure values, and vice versa. Am. J. Epidemiol..

[41] Tobin MD, Sheehan NA, Scurrah KJ, Burton PR (2005). Adjusting for treatment effects in studies of quantitative traits: Antihypertensive therapy and systolic blood pressure. Stat. Med..

[42] Palmer TM (2012). Using multiple genetic variants as instrumental variables for modifiable risk factors. Stat. Methods Med. Res..

[43] Hemani G, Tilling K, Davey Smith G (2017). Orienting the causal relationship between imprecisely measured traits using GWAS summary data. PLoS Genet..

[44] Lee SH, Goddard ME, Wray NR, Visscher PM (2012). A better coefficient of determination for genetic profile analysis. Genetic Epidemiology.

[45] Teslovich TM (2010). Biological, clinical and population relevance of 95 loci for blood lipids. Nature.

[46] Bowden J (2017). A framework for the investigation of pleiotropy in two-sample summary data Mendelian randomization. Stat. Med..

[47] Del Greco MF, Minelli C, Sheehan NA, Thompson JR (2015). Detecting pleiotropy in Mendelian randomisation studies with summary data and a continuous outcome. Stat. Med..

[48] Bowden J, Davey Smith G, Burgess S (2015). Mendelian randomization with invalid instruments: Effect estimation and bias detection through Egger regression. Int. J. Epidemiol..

[49] Bowden J (2016). Assessing the suitability of summary data for two-sample Mendelian randomization analyses using MR-Egger regression: The role of the I2 statistic. Int. J. Epidemiol..

[50] Verbanck M, Chen C-Y, Neale B, Do R (2018). Detection of widespread horizontal pleiotropy in causal relationships inferred from Mendelian randomization between complex traits and diseases. Nat. Genet..

[51] Rees JMB, Wood AM, Dudbridge F, Burgess S (2019). Robust methods in Mendelian randomization via penalization of heterogeneous causal estimates. PLoS ONE.

[52] Stone M (1974). Cross-validatory choice and assessment of statistical predictions. J. R. Stat. Soc. Ser. B.

[53] Corbin LJ (2016). BMI as a modifiable risk factor for type 2 diabetes: Refining and understanding causal estimates using mendelian randomization. Diabetes.

[54] Bowden J, Davey Smith G, Haycock PC, Burgess S (2016). Consistent estimation in Mendelian randomization with some invalid instruments using a weighted median estimator. Genet. Epidemiol..

[55] Hartwig FP, Davey Smith G, Bowden J (2017). Robust inference in summary data Mendelian randomization via the zero modal pleiotropy assumption. Int. J. Epidemiol..

[56] Qi, G. & Chatterjee, N. Mendelian randomization analysis using mixture models for robust and efficient estimation of causal effects. *Nat. Commun.* **10** Article 1941 (2019). 10.1038/s41467-019-09432-2 PMC6486646 31028273

[57] Rees JMB, Wood AM, Burgess S (2017). Extending the MR-Egger method for multivariable Mendelian randomization to correct for both measured and unmeasured pleiotropy. Stat. Med..

[58] StataCorp. Stata Statistical Software: Release 15. College Station, TX: StataCorp LLC. (2017).

[59] R Core Team. R: A language and environment for statistical computing. R Foundation for Statistical Computing, Vienna, Austria. https://www.R-project.org/. (2020).

[60] Hemani, G. *et al.* The MR-Base platform supports systematic causal inference across the human phenome. *Elife* **7** Article e34408 (2018). 10.7554/eLife.34408 PMC5976434 29846171

[61] Yavorska OO, Burgess S (2017). MendelianRandomization: An R package for performing Mendelian randomization analyses using summarized data. Int. J. Epidemiol..

