# Supplementary material for: Atrial fibrillation and left atrial size and function: a Mendelian randomization study
Source: Sci Rep. 2021 Apr 19;11:8431. doi: 10.1038/s41598-021-87859-8 (PMC8055882; doi:10.1038/s41598-021-87859-8)
Supplement: Supplementary file 2 — Supplementary Information 2. [file 41598_2021_87859_MOESM2_ESM.pdf]

# Atrial Fibrillation and Left Atrial size and function

A Mendelian randomization study

## Supplementary Information

Yordi J. van de Vegte[1] †, Bsc, Joylene E. Siland[1] †, Bsc, Michiel Rienstra[1], MD, PhD, Pim van der Harst[1,2,3], MD, PhD

[1] Department of Cardiology, University of Groningen, University Medical Center Groningen, Groningen, The Netherlands. P.O. Box 30.001, 9700 RB Groningen, The Netherlands.

[2] University of Groningen, University Medical Center Groningen, Department of Genetics, Groningen, The Netherlands

[3] University Medical Center Utrecht, Department of Heart and Lungs, University of Utrecht, Utrecht, The Netherlands

**Table of contents supplementary data**

**Table 1.** Disease definitions used in the UK Biobank study

**Figure 1.** Flowchart selection study sample

**Figure 2.** Scatter- (A) and forestplot (B) of the MR between AF and LA max

**Figure 3.** Scatter- (A) and forestplot (B) of the MR between AF and LA max indexed

**Figure 4.** Scatter- (A) and forestplot (B) of the MR between AF and LA min

**Figure 5.** Scatter- (A) and forestplot (B) of the MR between AF and LA min indexed

**Figure 6.** Scatter- (A) and forestplot (B) of the MR between AF and LA stroke volume

**Figure 7.** Scatter- (A) and forestplot (B) of the MR between AF and LA stroke volume indexed

**Figure 8.** Scatter- (A) and forestplot (B) of the MR between AF and LA ejection fraction

**Figure 9.** Leave-one-out MR-IVW (A) and MR-Egger (B) analyses of the MR between AF and LA max

**Figure 10.** Leave-one-out MR-IVW (A) and MR-Egger (B) analyses of the MR between AF and LA max indexed

**Figure 11.** Leave-one-out MR-IVW (A) and MR-Egger (B) analyses of the MR between AF and LA min

**Figure 12.** Leave-one-out MR-IVW (A) and MR-Egger (B) analyses of the MR between AF and LA min indexed

**Figure 13.** Leave-one-out MR-IVW (A) and MR-Egger (B) analyses of the MR between AF and LA stroke volume

**Figure 14.** Leave-one-out MR-IVW (A) and MR-Egger (B) analyses of the MR between AF and LA stroke volume indexed

**Figure 15.** Leave-one-out MR-IVW (A) and MR-Egger (B) analyses of the MR between AF and LA ejection fraction

**Figure 16.** Histogram of LA dimension distributions per AF increasing T allele of rs67249485

**Table of contents of online data**

**Table 1.** Single genetic variant exposure, outcome and exposure-outcome associations between atrial fibrillation and LA size and function.

**Table 2.** Results of the leave-one-out Mendelian randomization analyses between AF and LA size and function in 4,274 individuals with cardiovascular magnetic resonance data, excluding those with prevalent atrial fibrillation.

**Supplementary Table 1.** Disease definitions used in the UK Biobank study

| Trait | ICD-10 | ICD-9 | OPCS-4 | Touchscreen, disease | Touchscreen, medication | Non-cancer illness, self-reported (n_20002) | Operation code, self-reported (n_20004) |
|---|---|---|---|---|---|---|---|
| Heart failure | I42, I43, I50, I110, I255, I130, I132, O903, O994 | 425 | | | | 1079, 1588 | 1098 |
| Atrial fibrillation/ flutter | I48 | 4273 | K621, K622, K623 | | | 1471, 1483 | |
| Mitral valve disease | I34, I511 | 394,424 | | | | 1488, 1489, 1584, 1585 | 1100 |
| Pulmonary hypertension | I270 | 4160 | X82 | | | | |
| Valve Surgery | | | K25,K26,K27,K28,K29, K30,K31,K33,K34 | | | | 1097, 1099, 1100, 1101 |
| Type 2 Diabetes | E11 | 250 | | | | 1223 | |
| Hypertension | I10, I11, I12, I13, I15, O10 | 401, 402, 403, 404, 405 | | n_6150_=4 | n_6153_=2,n_6177_=2 | 1065, 1072 | |

*Variable definitions constructed using ICD-9, ICD-10 and OPCS-4 codes as well as self-reported data fields. Self-reported data fields include those obtained from the touchscreen questionnaire (on disease and medication) and those obtained during the interview with a trained nurse (n_20002 and n_20004). Abbreviations: ICD, International Classification of Diseases; OPCS, Office of Population, Censuses and Surveys: Classification of interventions and Procedures*

**Supplementary Figure 1.** Flowchart selection study sample

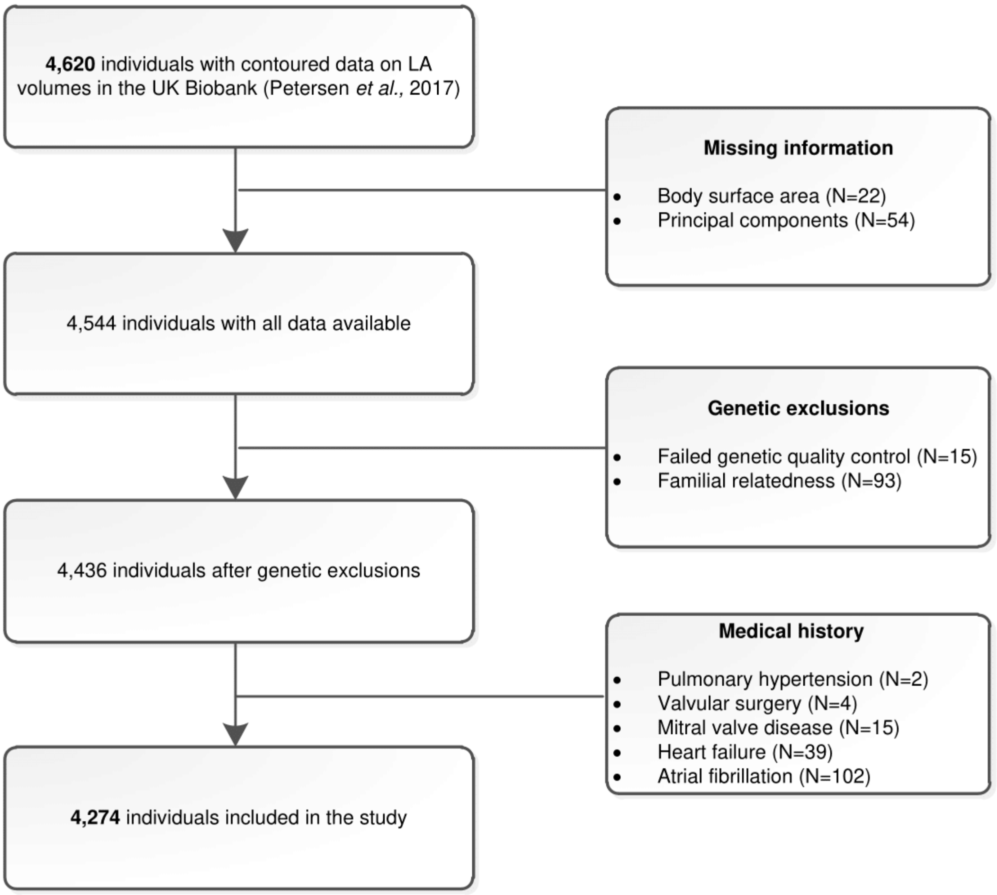


*Flowchart of the study population selection. Individuals were excluded in case of missing information on body surface area or any covariates (please see below), failure of genetic quality control (including heterozygosity, high missingness and a discrepancy between reported and inferred gender), familial relatedness, or a medical history of , pulmonary hypertension, valvular surgery, mitral valve disease, heart failure or prevalent AF at the time of cardiovascular magnetic resonance imaging.*

**Supplementary Figure 2.** Scatter- (A) and forestplot (B) of the MR between AF and LA max

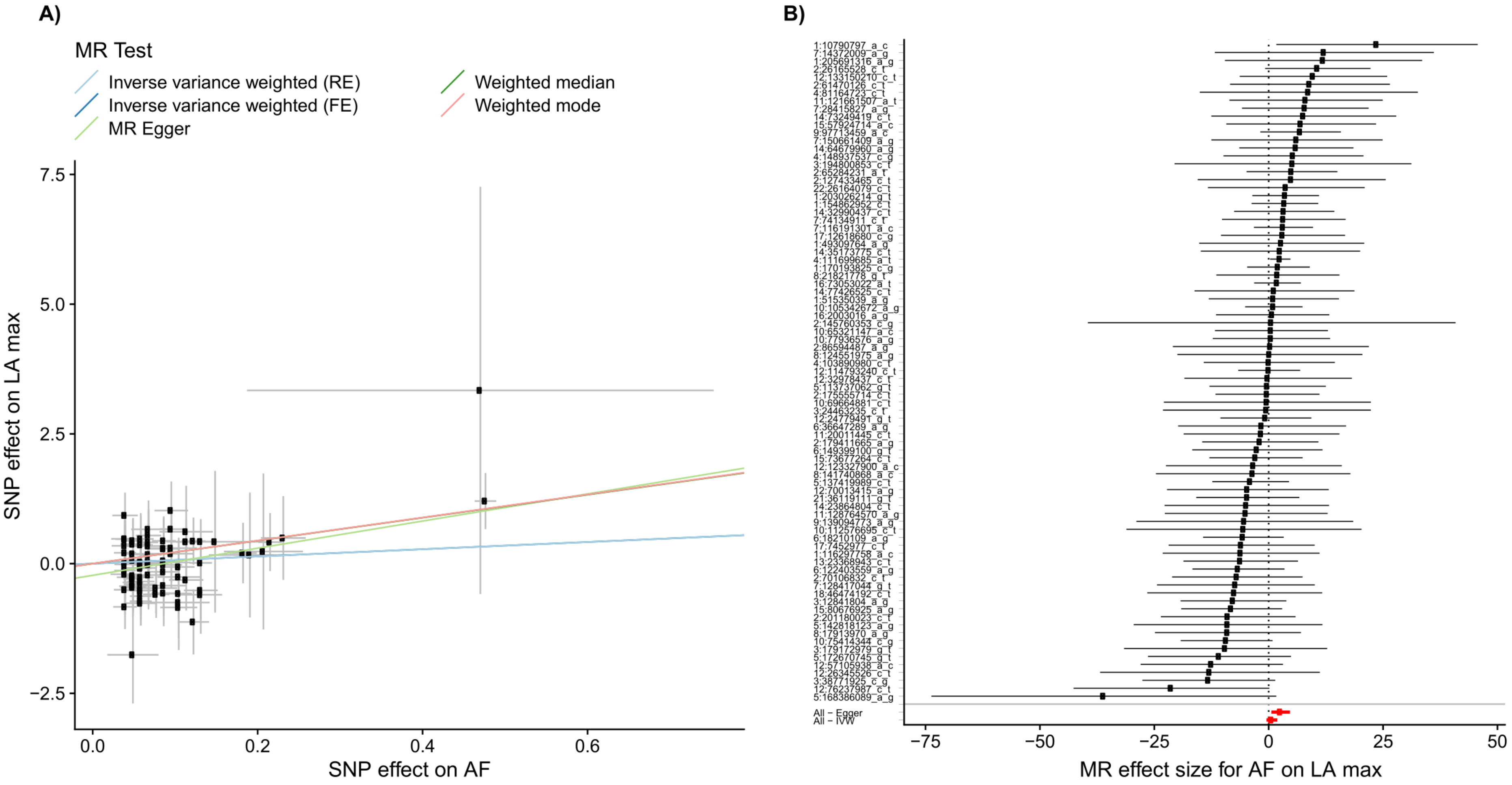


*A) Scatter plot including the MR estimates between AF and LA max. The variants' effect size and standard error on AF are displayed on the X-axis, the variants' effect size and standard error on LA max on the Y-axis. B) Forest plot of AF genetic variants associations with LA max. The Mendelian Randomization effect size and standard error of AF on LA max product are displayed on the X-axis. The different genetic variants for AF are listed on the Y-axis. AF = atrial fibrillation, LA max = maximal left atrial volume at the end of left ventricular systole, SNP = single nucleotide polymorphism, MR = Mendelian randomization.*

**Supplementary Figure 3.** Scatter- (A) and forestplot (B) of the MR between AF and indexed LA max

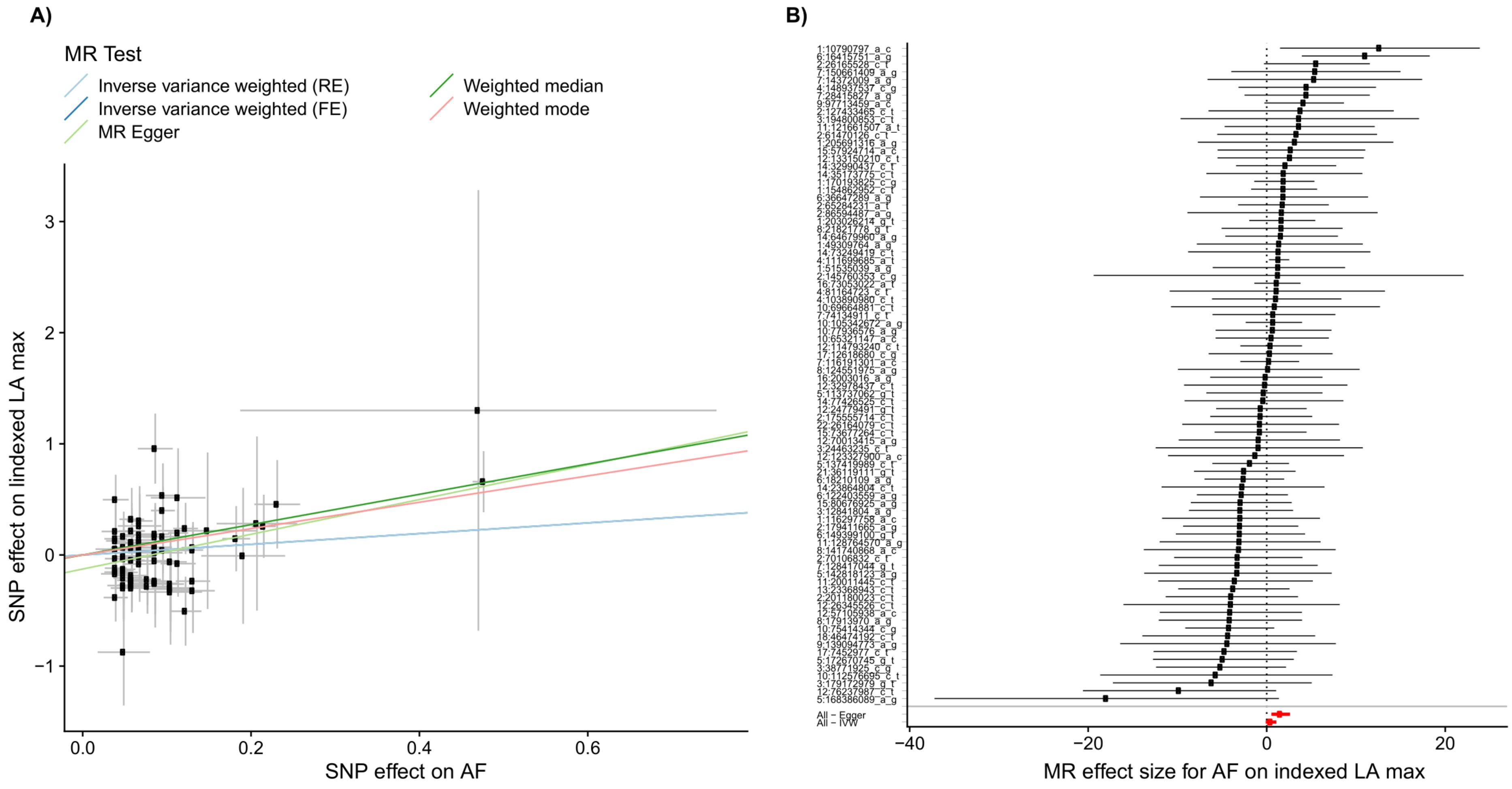


*A) Scatter plot including the MR estimates between AF and LA max indexed. The variants' effect size and standard error on AF are displayed on the X-axis, the variants' effect size and standard error on LA max indexed on the Y-axis. B) Forest plot of AF genetic variants associations with LA max indexed. The Mendelian Randomization effect size and standard error of AF on LA max indexed product are displayed on the X-axis. The different genetic variants for AF are listed on the Y-axis. AF = atrial fibrillation, LA max indexed = maximal left atrial volume at the end of left ventricular systole indexed for body surface area, SNP = single nucleotide polymorphism, MR = Mendelian randomization.*

**Supplementary Figure 4.** Scatter- (A) and forestplot (B) of the MR between AF and LA min

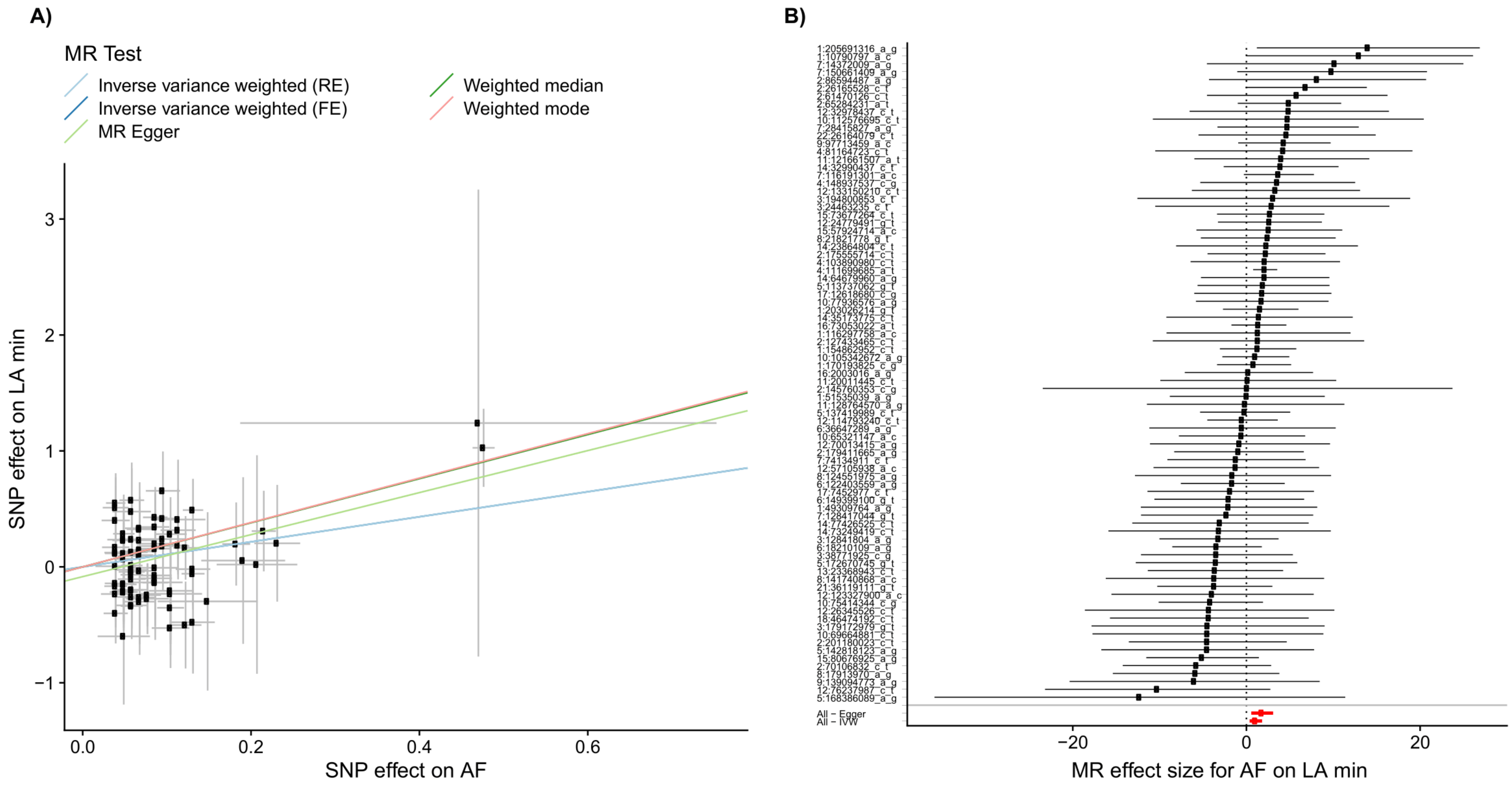


*A) Scatter plot including the MR estimates between AF and LA min. The variants' effect size and standard error on AF are displayed on the X-axis, the variants' effect size and standard error on LA min on the Y-axis. B) Forest plot of AF genetic variants associations with LA min. The Mendelian Randomization effect size and standard error of AF on LA min product are displayed on the X-axis. The different genetic variants for AF are listed on the Y-axis. AF = atrial fibrillation, LA min = minimal left atrial volume at the end of left ventricular diastole, SNP = single nucleotide polymorphism, MR = Mendelian randomization.*

**Supplementary Figure 5.** Scatter- (A) and forestplot (B) of the MR between AF and indexed LA min

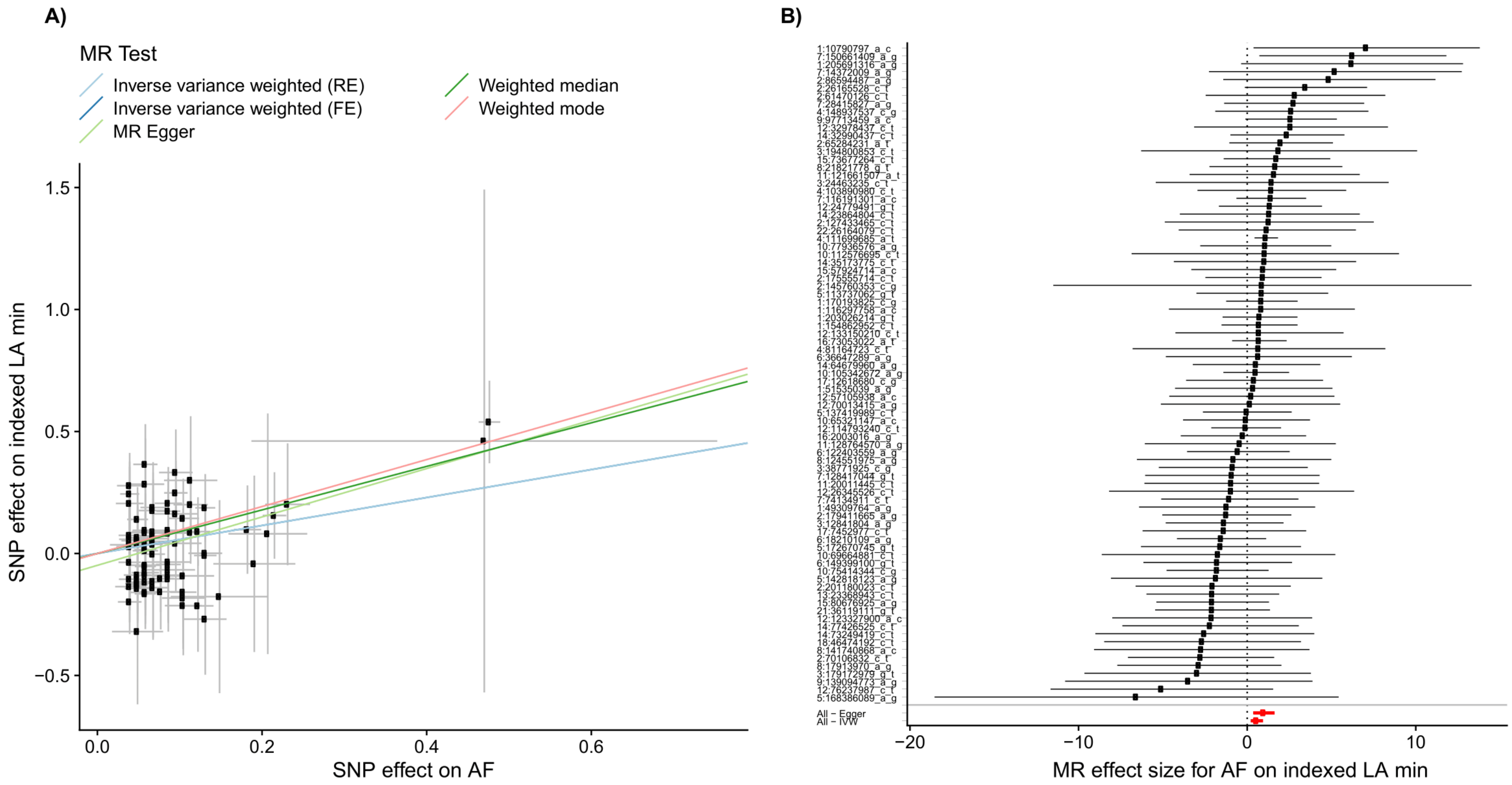


*A) Scatter plot including the MR estimates between AF and LA min indexed. The variants' effect size and standard error on AF are displayed on the X-axis, the variants' effect size and standard error on LA min indexed on the Y-axis. B) Forest plot of AF genetic variants associations with LA min indexed. The Mendelian Randomization effect size and standard error of AF on LA min indexed product are displayed on the X-axis. The different genetic variants for AF are listed on the Y-axis. AF = atrial fibrillation, LA min indexed= minimal left atrial volume at the end of left ventricular diastole, indexed for body surface area, SNP = single nucleotide polymorphism, MR = Mendelian randomization.*

**Supplementary Figure 6.** Scatter- (A) and forestplot (B) of the MR between AF and LA stroke volume

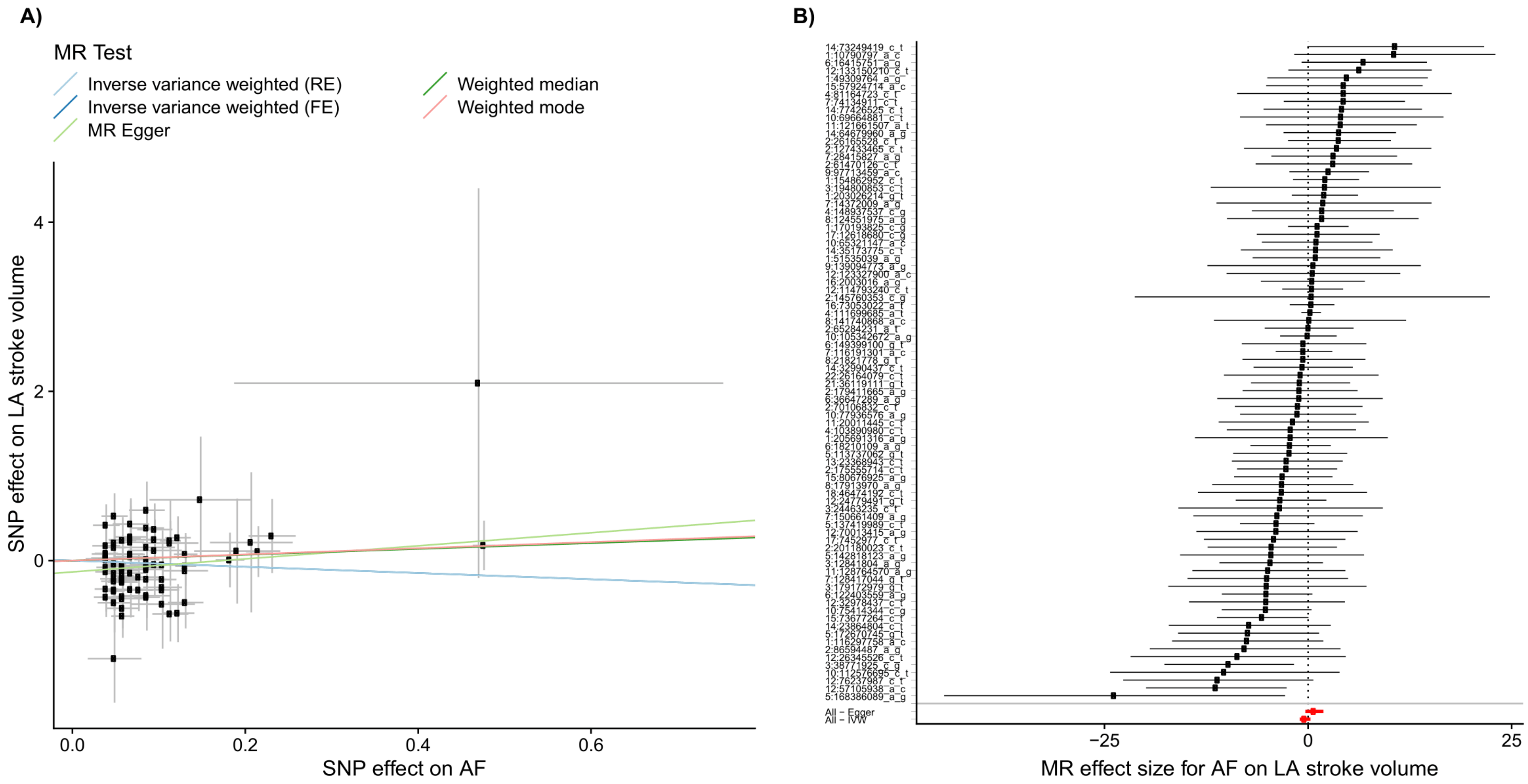


*A) Scatter plot including the MR estimates between AF and LA stroke volume. The variants' effect size and standard error on AF are displayed on the X-axis, the variants' effect size and standard error on LA stroke volume on the Y-axis. B) Forest plot of AF genetic variants associations with LA stroke volume. The Mendelian Randomization effect size and standard error of AF on LA stroke volume product are displayed on the X-axis. The different genetic variants for AF are listed on the Y-axis. AF = atrial fibrillation, LA stroke volume = maximal minus minimal left atrial volume SNP = single nucleotide polymorphism, MR = Mendelian randomization.*

**Supplementary Figure 7.** Scatter- (A) and forestplot (B) of the MR between AF and indexed LA stroke volume

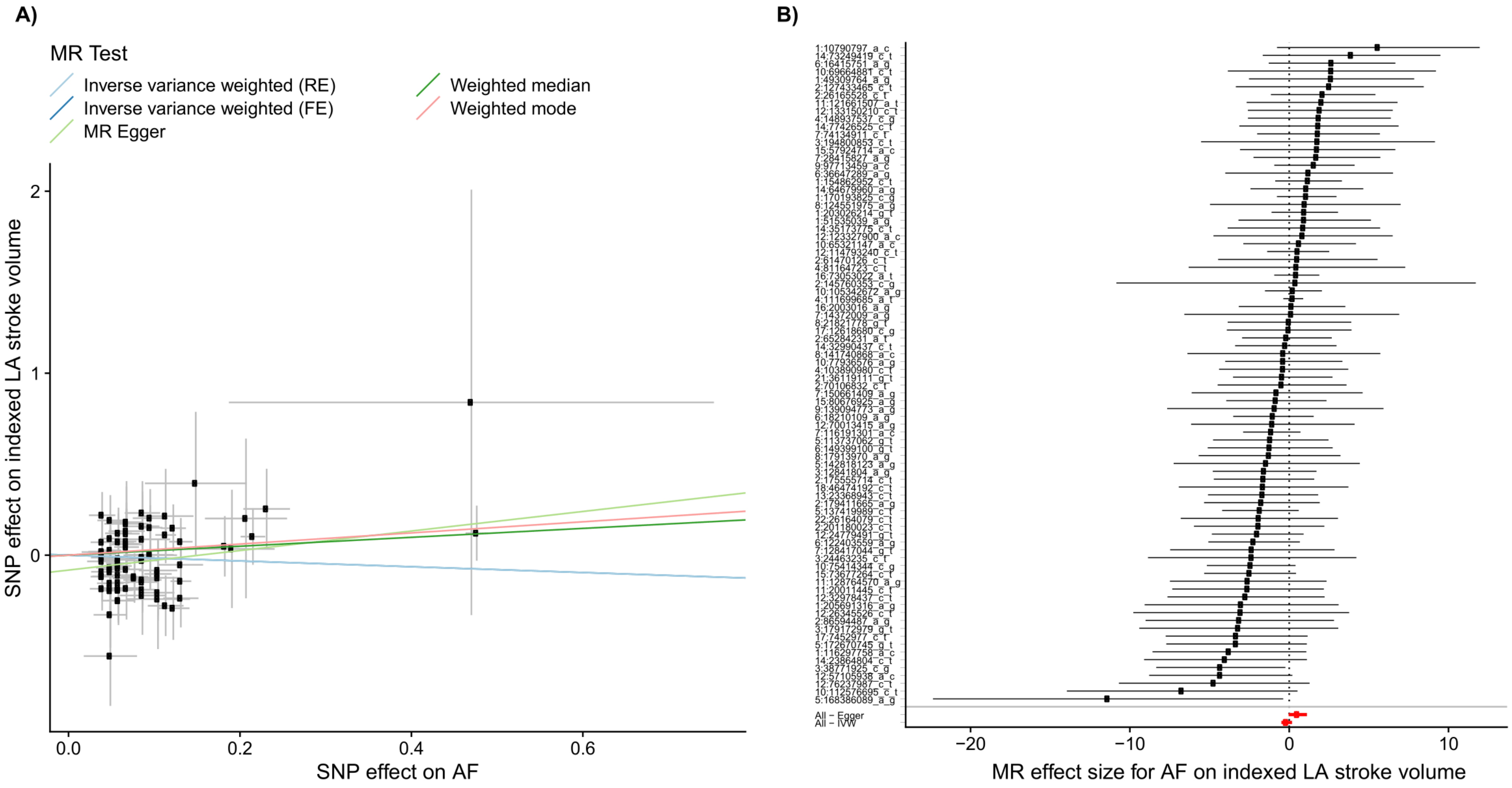


*A) Scatter plot including the MR estimates between AF and LA stroke volume indexed. The variants' effect size and standard error on AF are displayed on the X-axis, the variants' effect size and standard error on LA stroke volume indexed on the Y-axis. B) Forest plot of AF genetic variants associations with LA stroke volume indexed. The Mendelian Randomization effect size and standard error of AF on LA stroke volume indexed product are displayed on the X-axis. The different genetic variants for AF are listed on the Y-axis. AF = atrial fibrillation, LA stroke volume = maximal minus minimal left atrial volume, indexed for body surface area, SNP = single nucleotide polymorphism, MR = Mendelian randomization.*

**Supplementary Figure 8.** Scatter- (A) and forestplot (B) of the MR between AF and LA ejection fraction

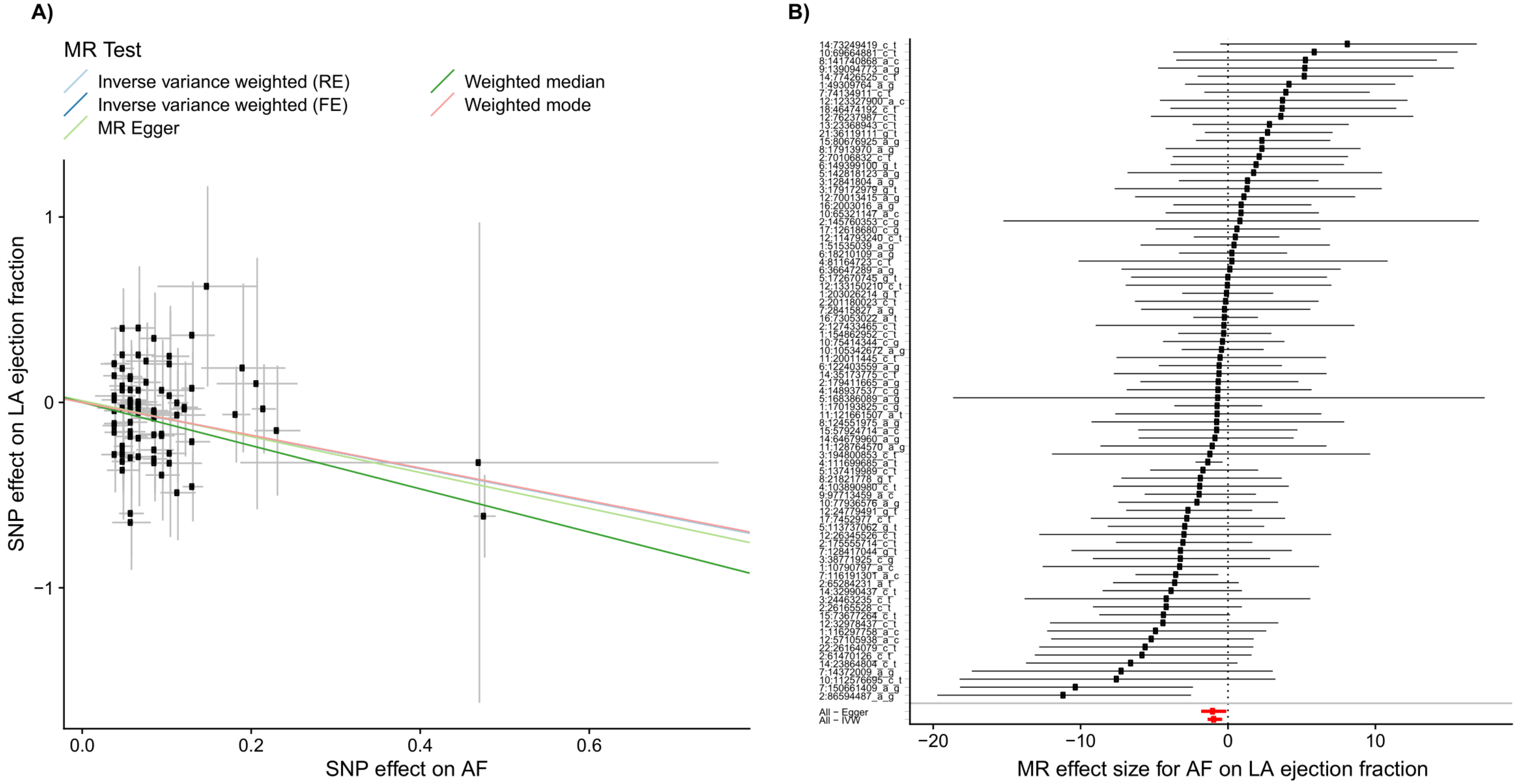


*A) Scatter plot including the MR estimates between AF and LA ejection fraction. The variants' effect size and standard error on AF are displayed on the X-axis, the variants' effect size and standard error on LA ejection fraction on the Y-axis. B) Forest plot of AF genetic variants associations with LA ejection fraction. The Mendelian Randomization effect size and standard error of AF on LA ejection fraction product are displayed on the X-axis. The different genetic variants for AF are listed on the Y-axis. AF = atrial fibrillation, LA ejection fraction = maximal minus minimal left atrial volume divided by maximal atrial volume, SNP = single nucleotide polymorphism, MR = Mendelian randomization.*

**Supplementary Figure 9.** Leave-one-out MR-IVW (A) and MR-Egger (B) analyses of the MR between AF and LA max

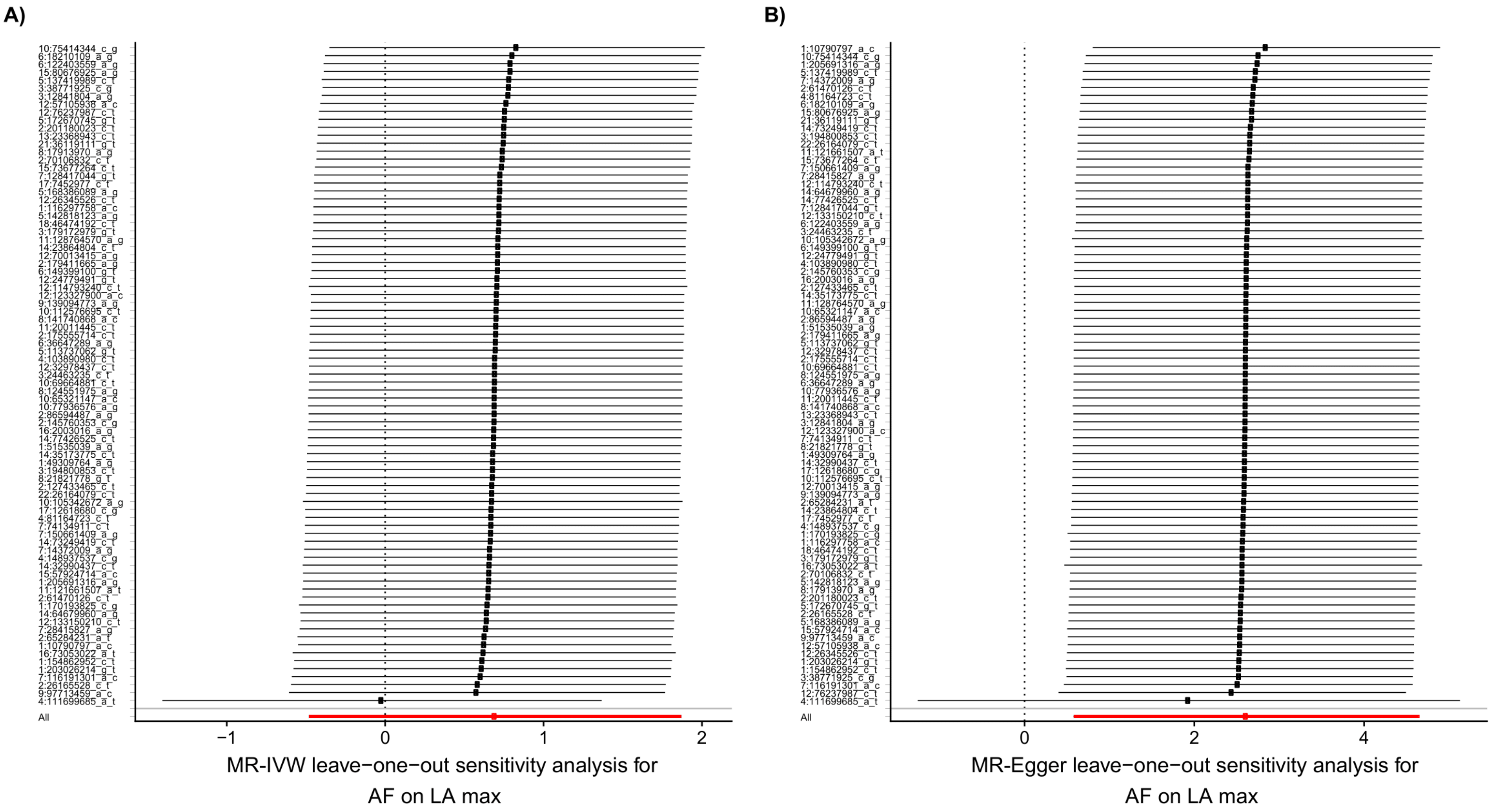


*Figure with results of the leave-one-out MR-IVW (A) and MR-Egger (B) analyses of the MR between AF and LA max. The Mendelian Randomization effect size and standard error of the MR-IVW (A) and MR-Egger (B) estimates of AF on LA max are displayed on the X-axis. The Y-axis displays the excluded genetic variant per MR estimate. AF = atrial fibrillation, LA max = maximal left atrial volume at the end of left ventricular systole, MR = Mendelian randomization, IVW = inverse variance weighted.*

**Supplementary Figure 10.** Leave-one-out MR-IVW (A) and MR-Egger (B) analyses of the MR between AF and indexed LA max

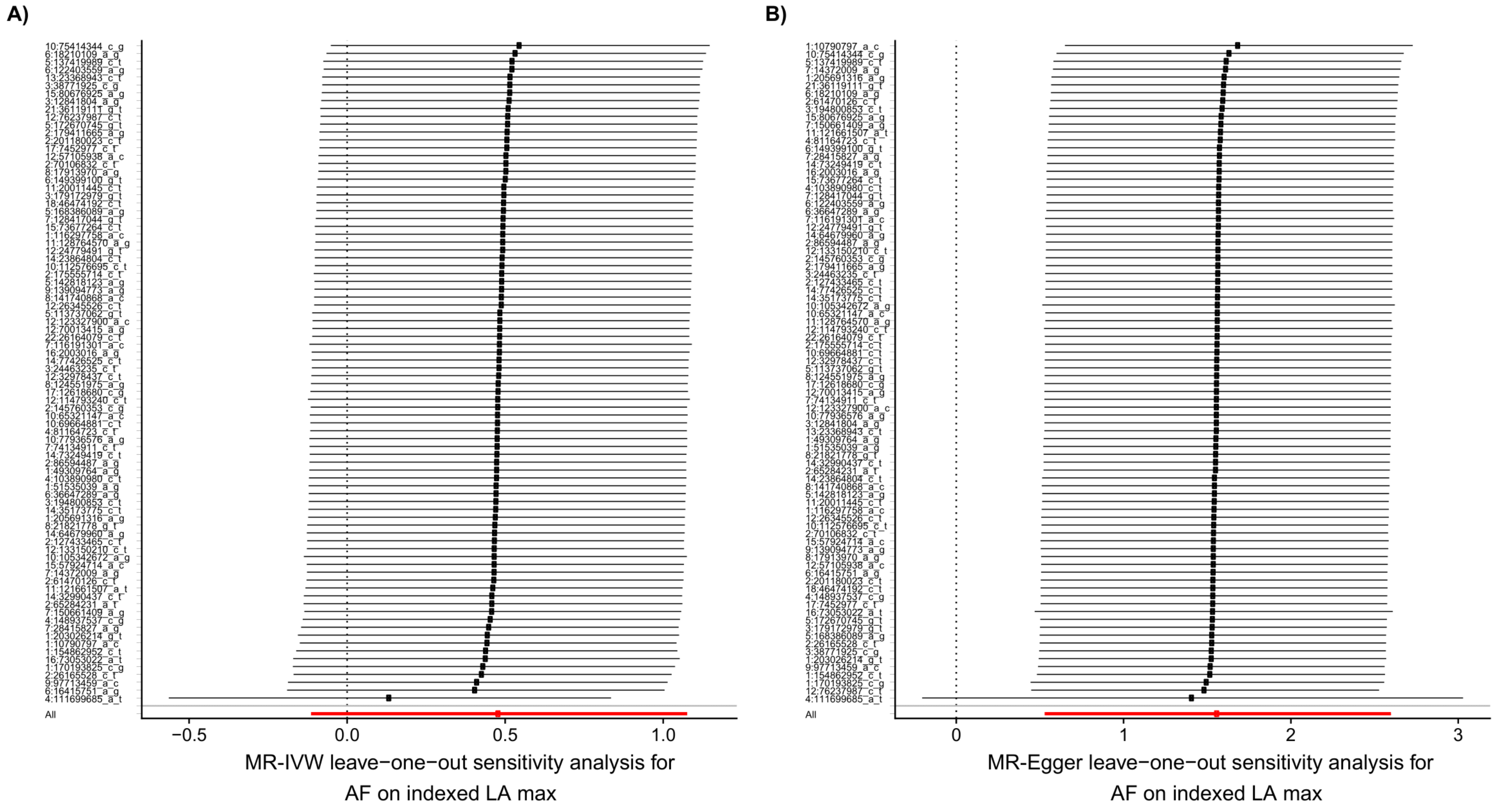


*Figure with results of the leave-one-out MR-IVW (A) and MR-Egger (B) analyses of the MR between AF and LA max indexed. The Mendelian Randomization effect size and standard error of the MR-IVW (A) and MR-Egger (B) estimates of AF on LA max indexed are displayed on the X-axis. The Y-axis displays the excluded genetic variant per MR estimate. AF = atrial fibrillation, LA max indexed = maximal left atrial volume at the end of left ventricular systole, indexed for body surface area, MR = Mendelian randomization, IVW = inverse variance weighted.*

**Supplementary Figure 11.** Leave-one-out MR-IVW (A) and MR-Egger (B) analyses of the MR between AF and LA min

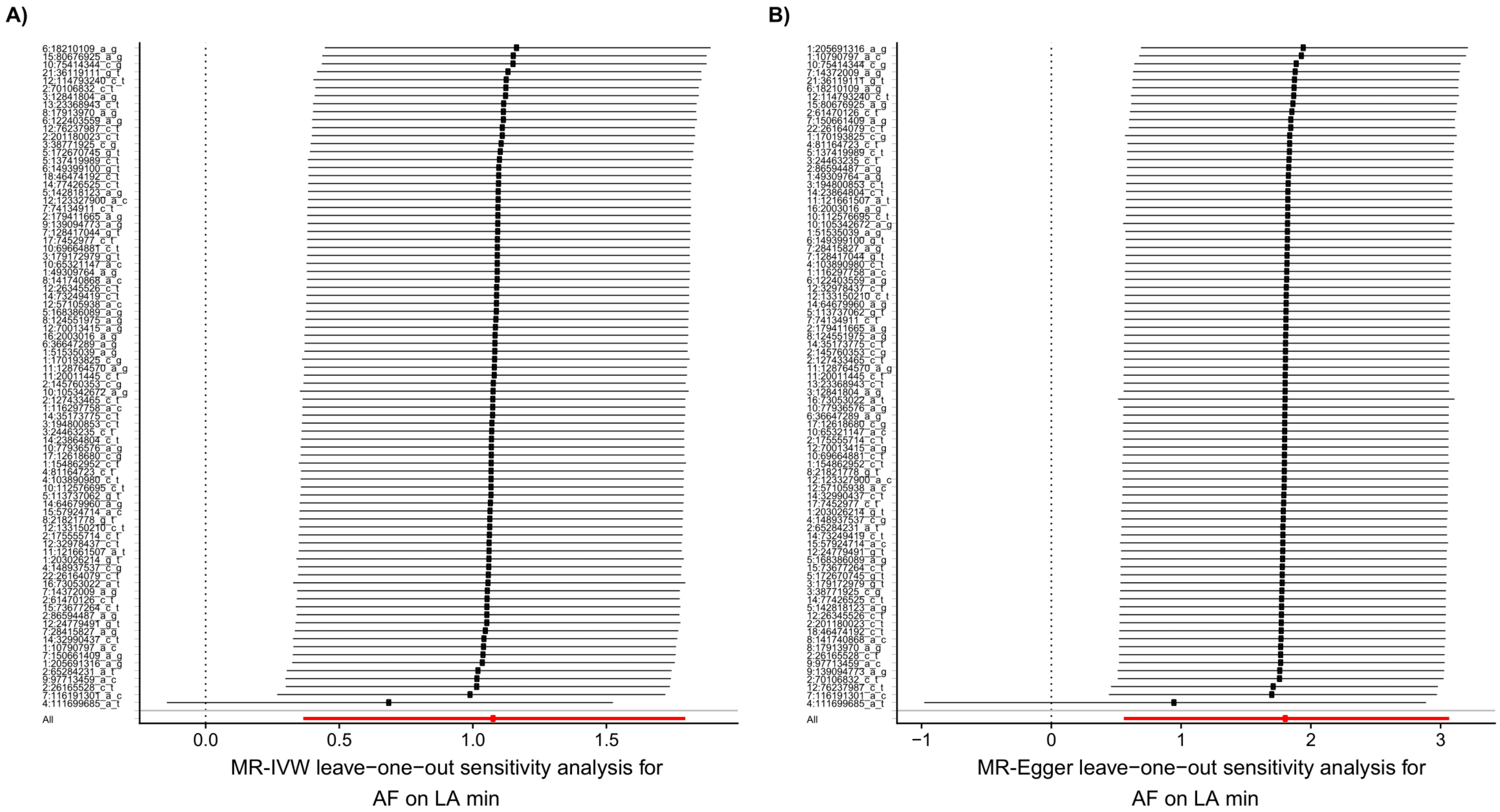


*Figure with results of the leave-one-out MR-IVW (A) and MR-Egger (B) analyses of the MR between AF and LA min. The Mendelian Randomization effect size and standard error of the MR-IVW (A) and MR-Egger (B) estimates of AF on LA min are displayed on the X-axis. The Y-axis displays the excluded genetic variant per MR estimate. AF = atrial fibrillation, LA min = minimal left atrial volume at the end of left ventricular diastole, MR = Mendelian randomization, IVW = inverse variance weighted.*

**Supplementary Figure 12.** Leave-one-out MR-IVW (A) and MR-Egger (B) analyses of the MR between AF and indexed LA min

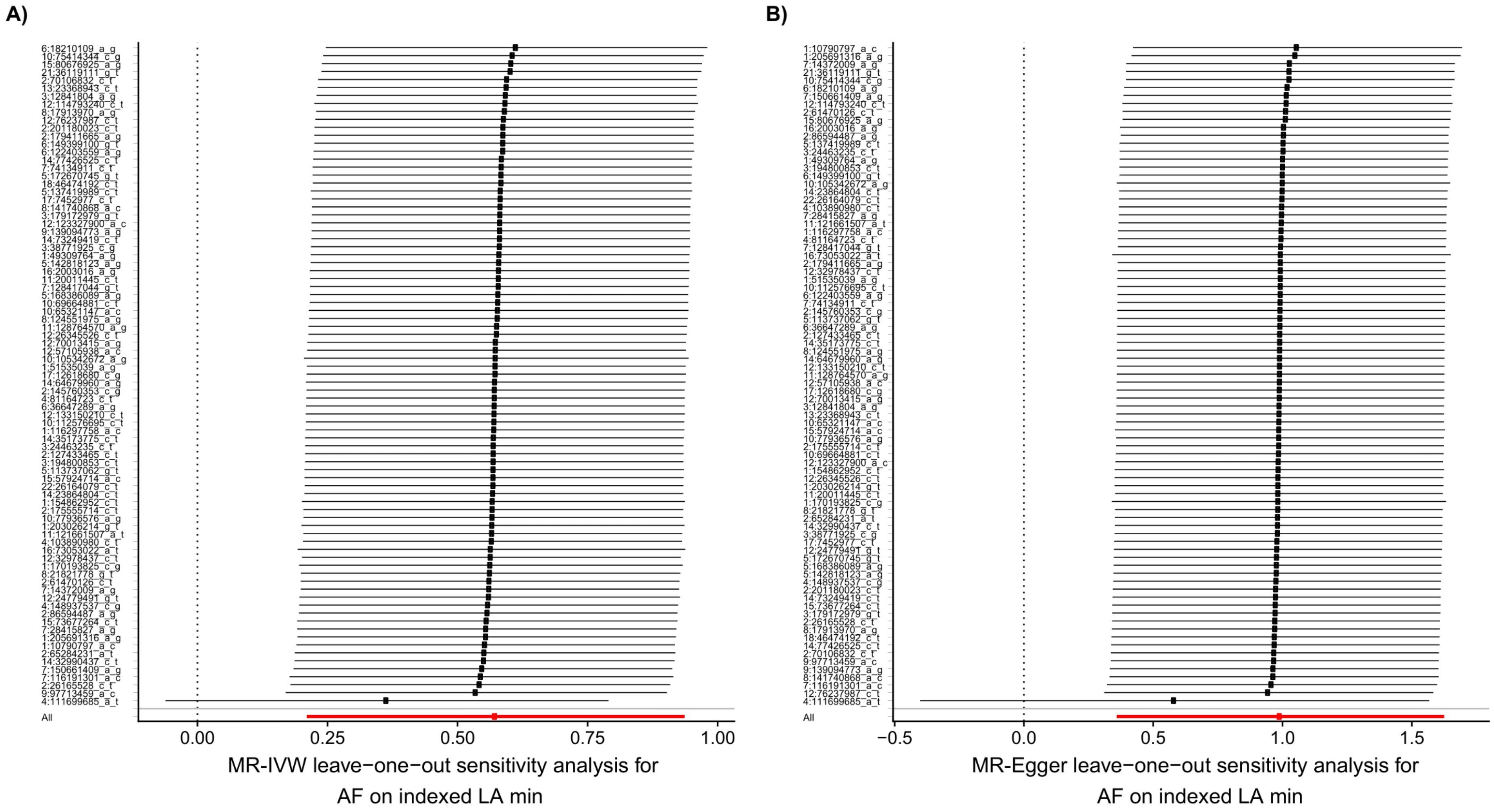


*Figure with results of the leave-one-out MR-IVW (A) and MR-Egger (B) analyses of the MR between AF and LA min indexed. The Mendelian Randomization effect size and standard error of the MR-IVW (A) and MR-Egger (B) estimates of AF on LA min indexed are displayed on the X-axis. The Y-axis displays the excluded genetic variant per MR estimate. AF = atrial fibrillation, LA min indexed = minimal left atrial volume at the end of left ventricular diastole, indexed for body surface area, MR = Mendelian randomization, IVW = inverse variance weighted.*

**Supplementary Figure 13.** Leave-one-out MR-IVW (A) and MR-Egger (B) analyses of the MR between AF and LA stroke volume

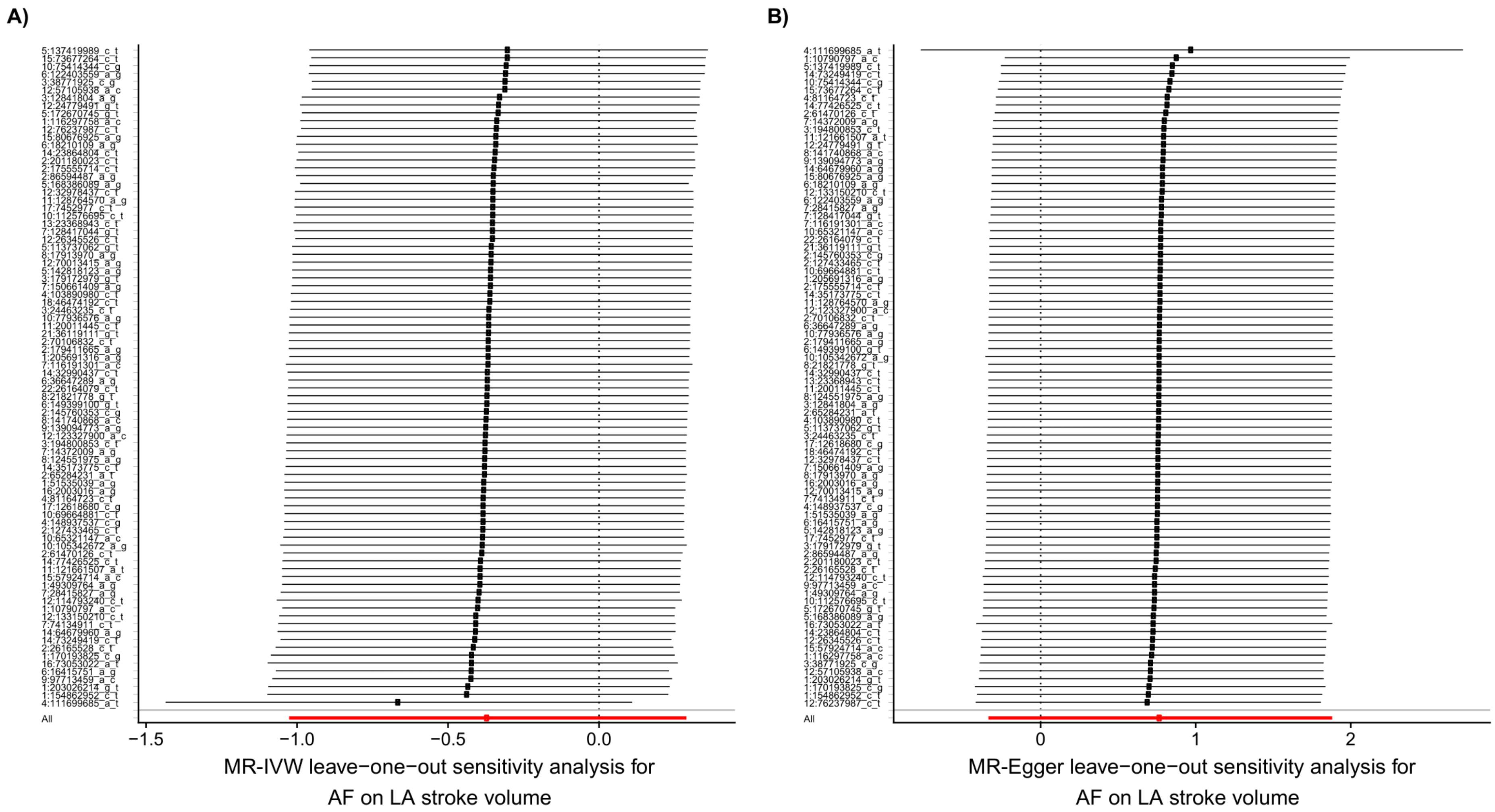


*Figure with results of the leave-one-out MR-IVW (A) and MR-Egger (B) analyses of the MR between AF and LA stroke volume. The Mendelian Randomization effect size and standard error of the MR-IVW (A) and MR-Egger (B) estimates of AF on LA stroke volume are displayed on the X-axis. The Y-axis displays the excluded genetic variant per MR estimate. AF = atrial fibrillation, LA stroke volume = maximal minus minimal left atrial volume, MR = Mendelian randomization, IVW = inverse variance weighted.*

**Supplementary Figure 14.** Leave-one-out MR-IVW (A) and MR-Egger (B) analyses of the MR between AF and indexed LA stroke volume

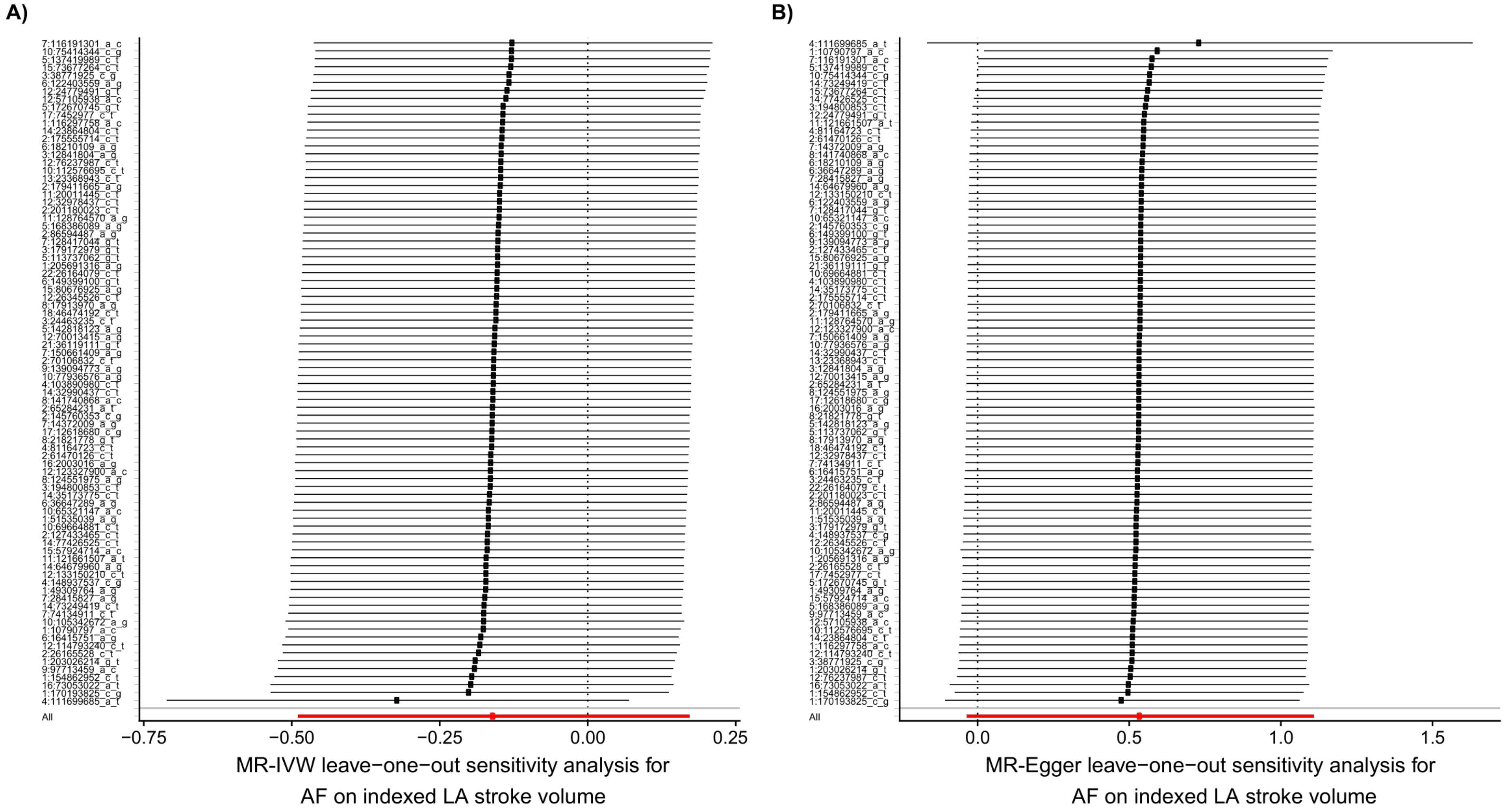


*Figure with results of the leave-one-out MR-IVW (A) and MR-Egger (B) analyses of the MR between AF and LA stroke volume indexed. The Mendelian Randomization effect size and standard error of the MR-IVW (A) and MR-Egger (B) estimates of AF on LA stroke volume indexed are displayed on the X-axis. The Y-axis displays the excluded genetic variant per MR estimate. AF = atrial fibrillation, LA stroke volume indexed = maximal minus minimal left atrial volume, indexed for body surface area, MR = Mendelian randomization, IVW = inverse variance weighted.*

**Supplementary Figure 15.** Leave-one-out MR-IVW (A) and MR-Egger (B) analyses of the MR between AF and LA ejection fraction

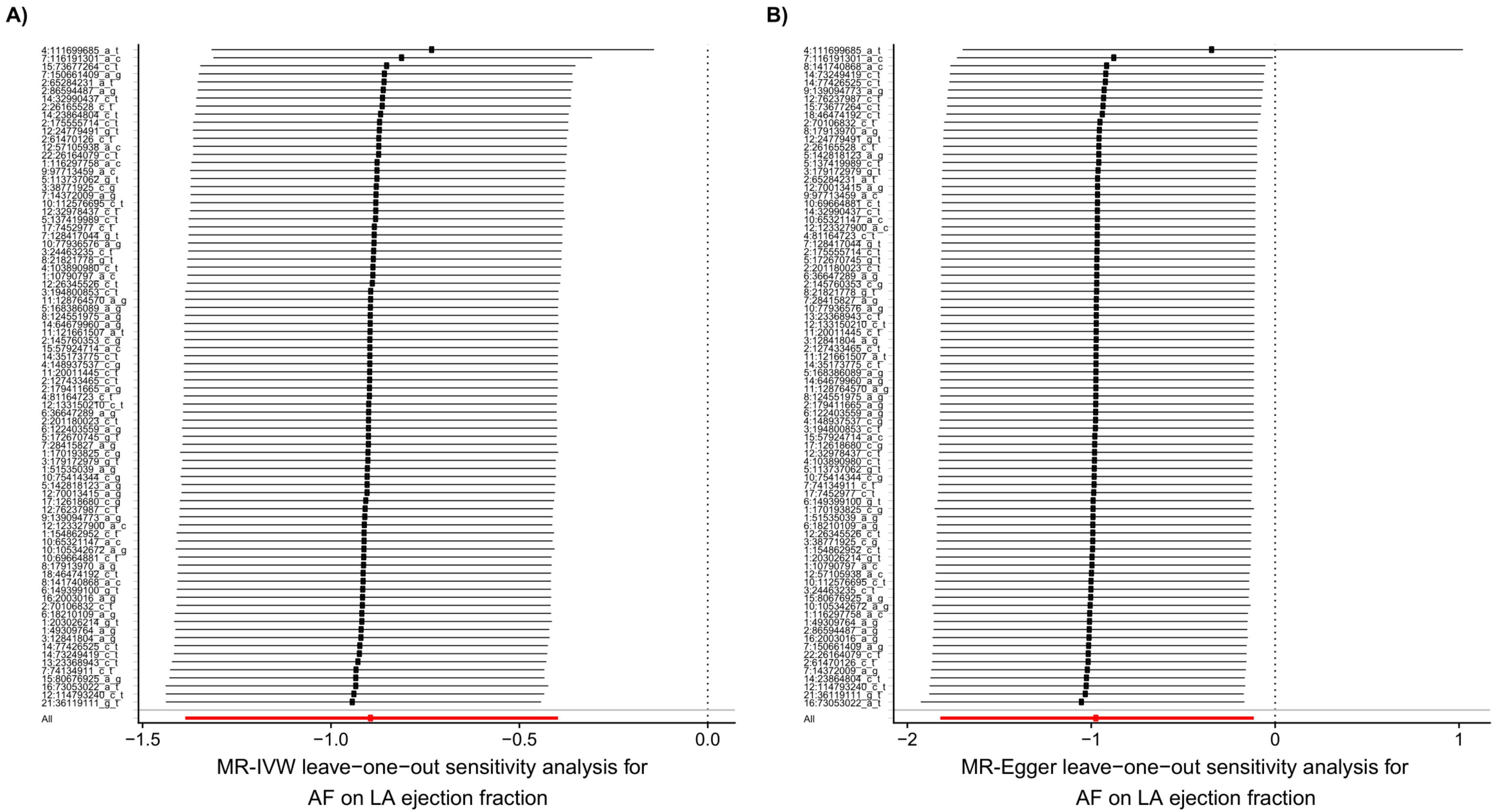


*Figure with results of the leave-one-out MR-IVW (A) and MR-Egger (B) analyses of the MR between AF and LA ejection fraction. The Mendelian Randomization effect size and standard error of the MR-IVW (A) and MR-Egger (B) estimates of AF on LA ejection fraction are displayed on the X-axis. The Y-axis displays the excluded genetic variant per MR estimate. AF = atrial fibrillation, LA ejection fraction = maximal minus minimal left atrial volume divided by the maximal volume, MR = Mendelian randomization, IVW = inverse variance weighted*

**Supplementary Figure 16.** Histogram of LA dimension distributions per AF increasing T allele of rs67249485

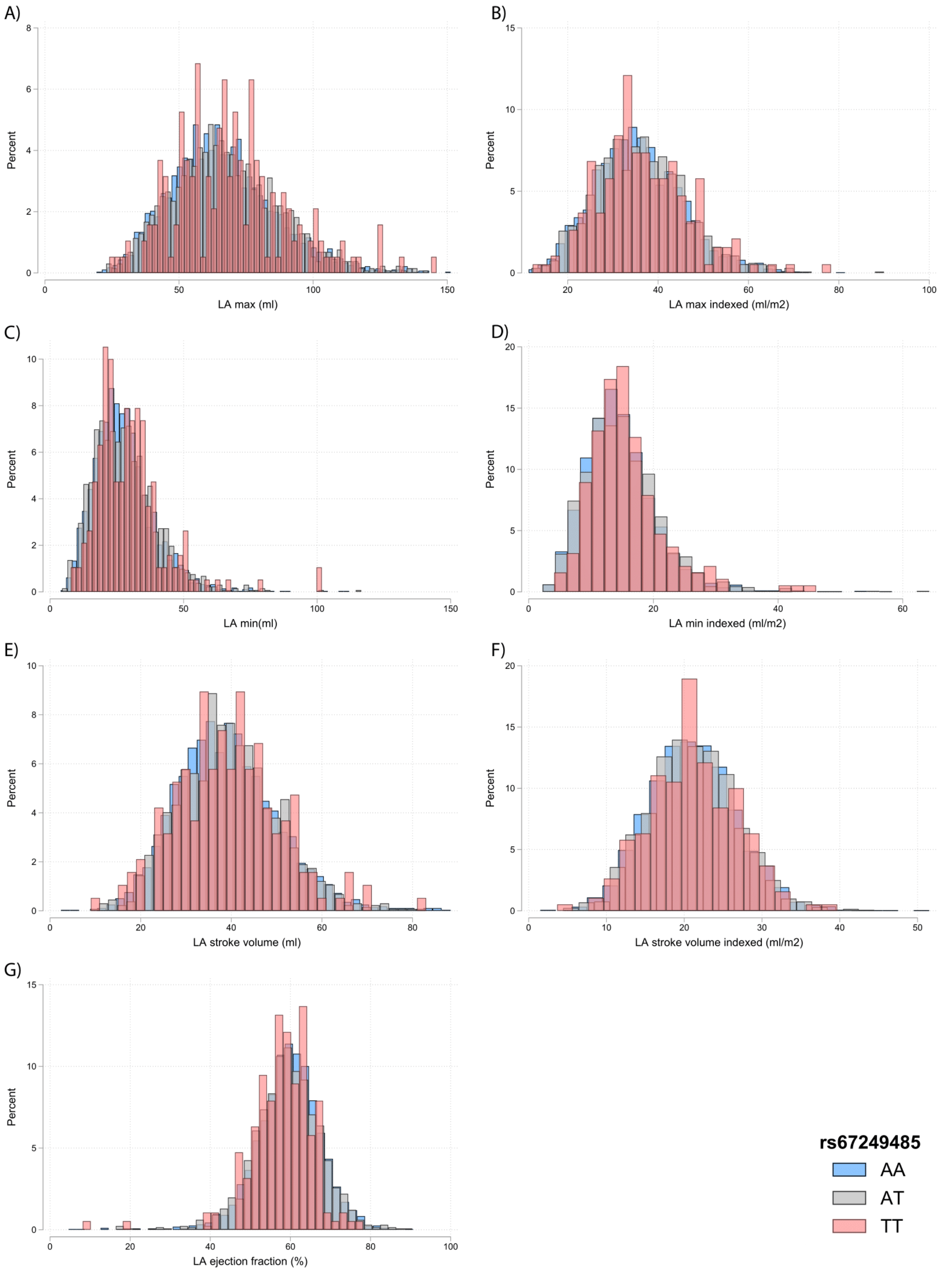


*Histograms of the distribution LA dimension distributions per AF increasing T allele of rs67249485.The information score of genetic variant rs67249485 is 0.993 and has a minor allele frequency of 0.205 in the population used in the current study.*
